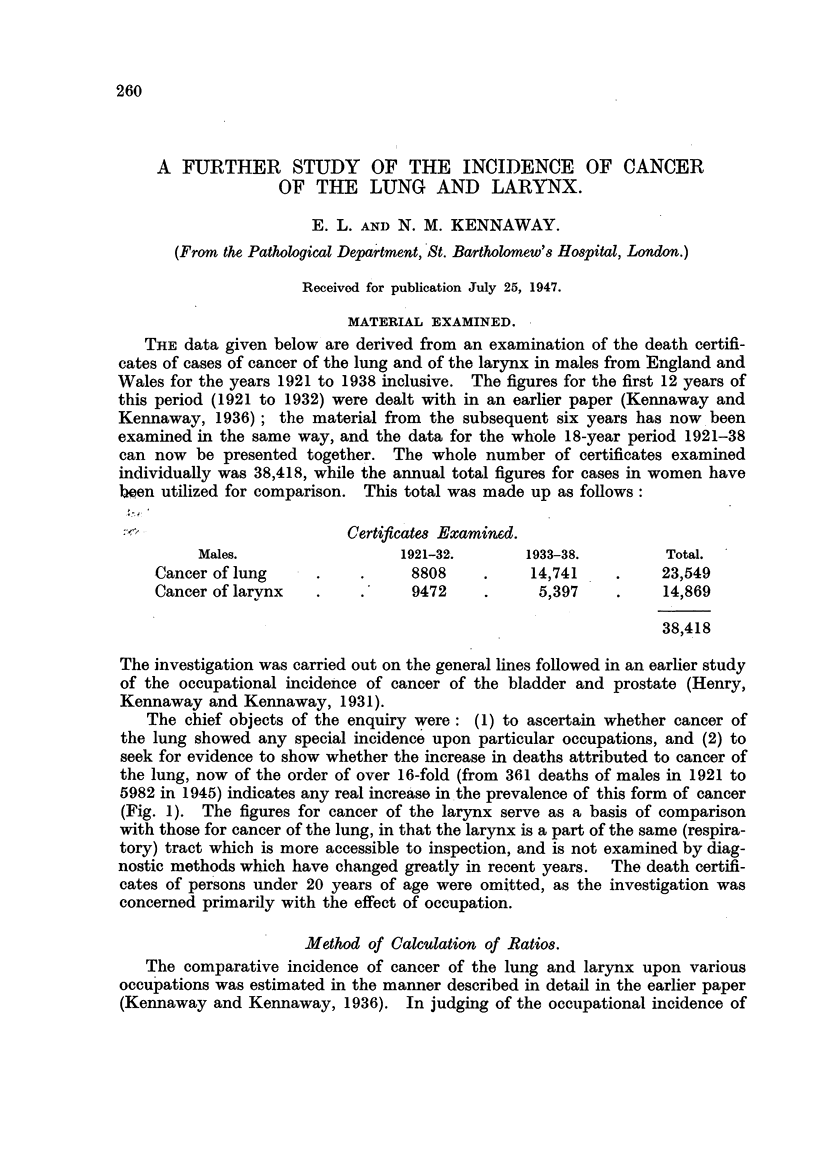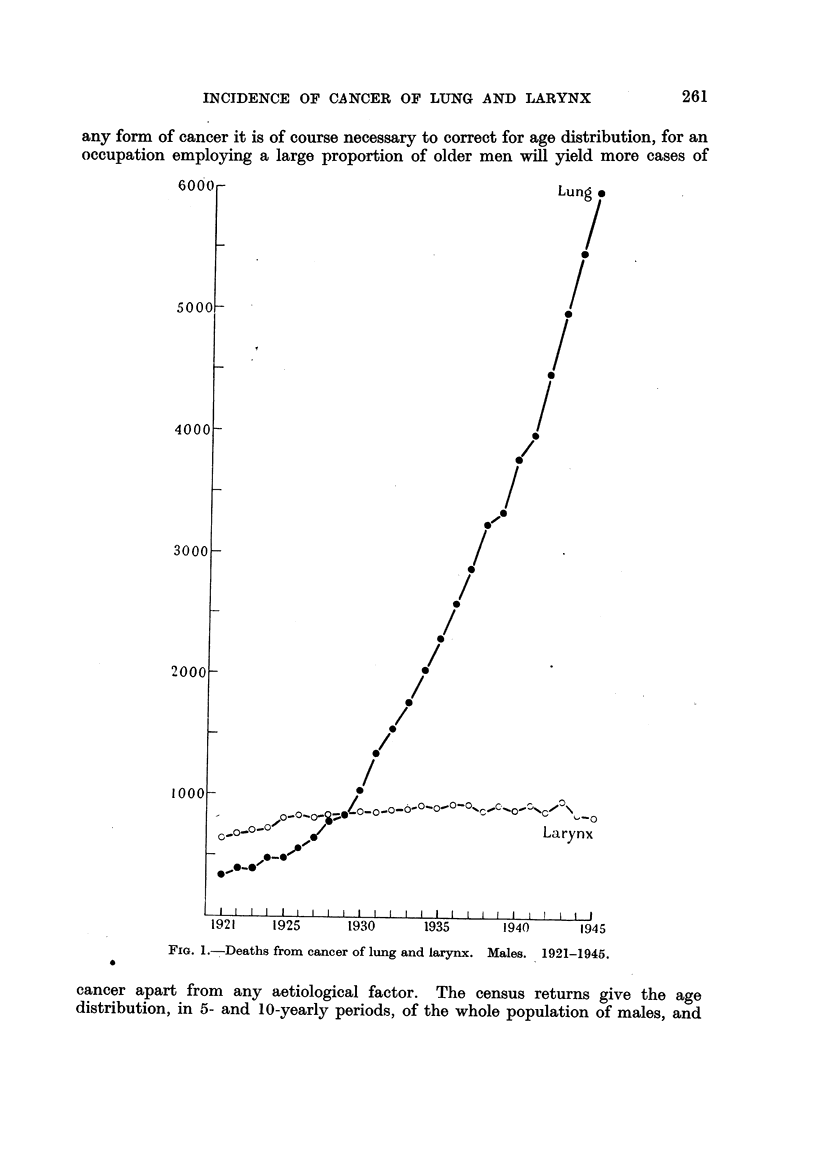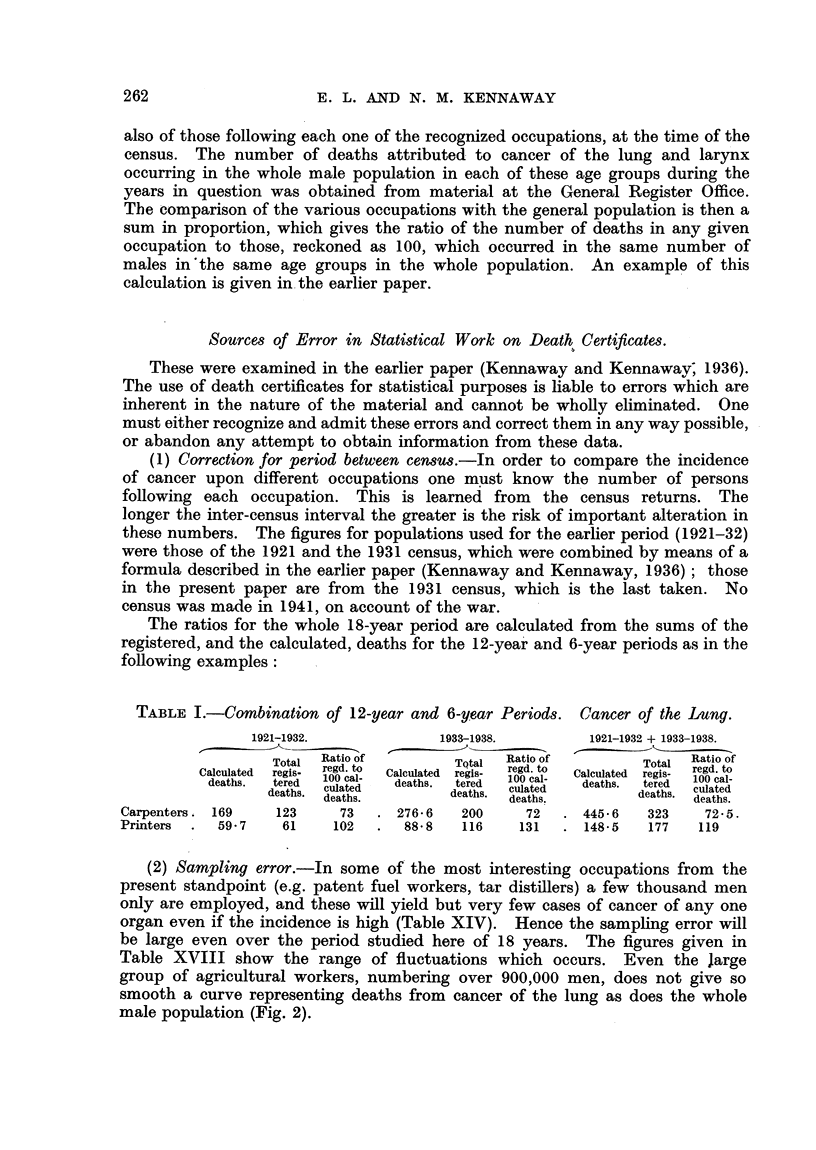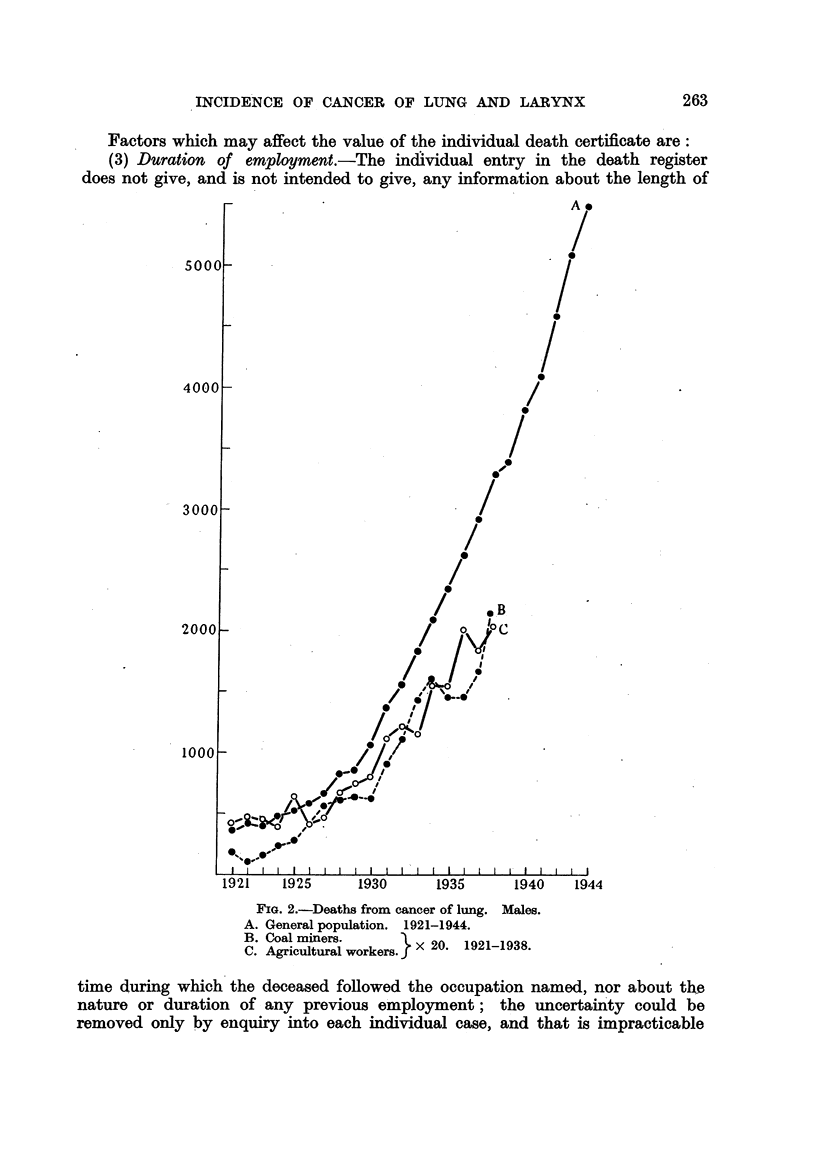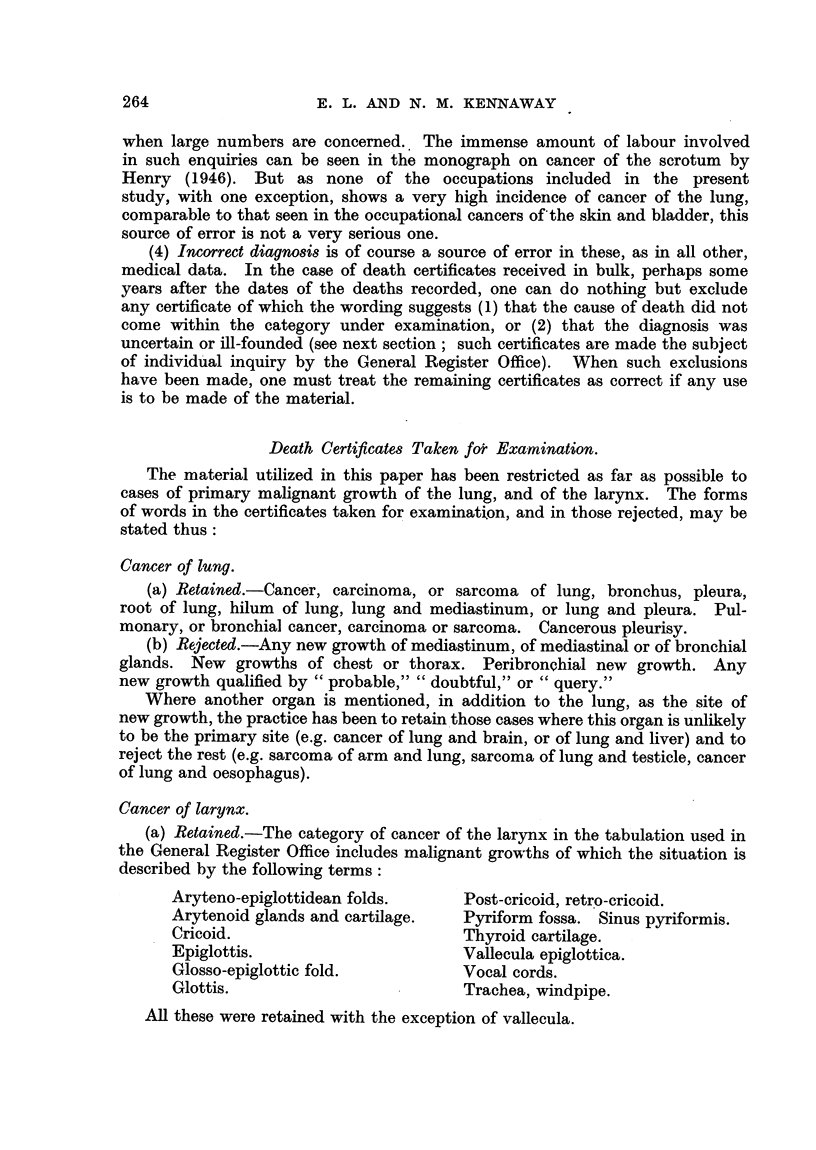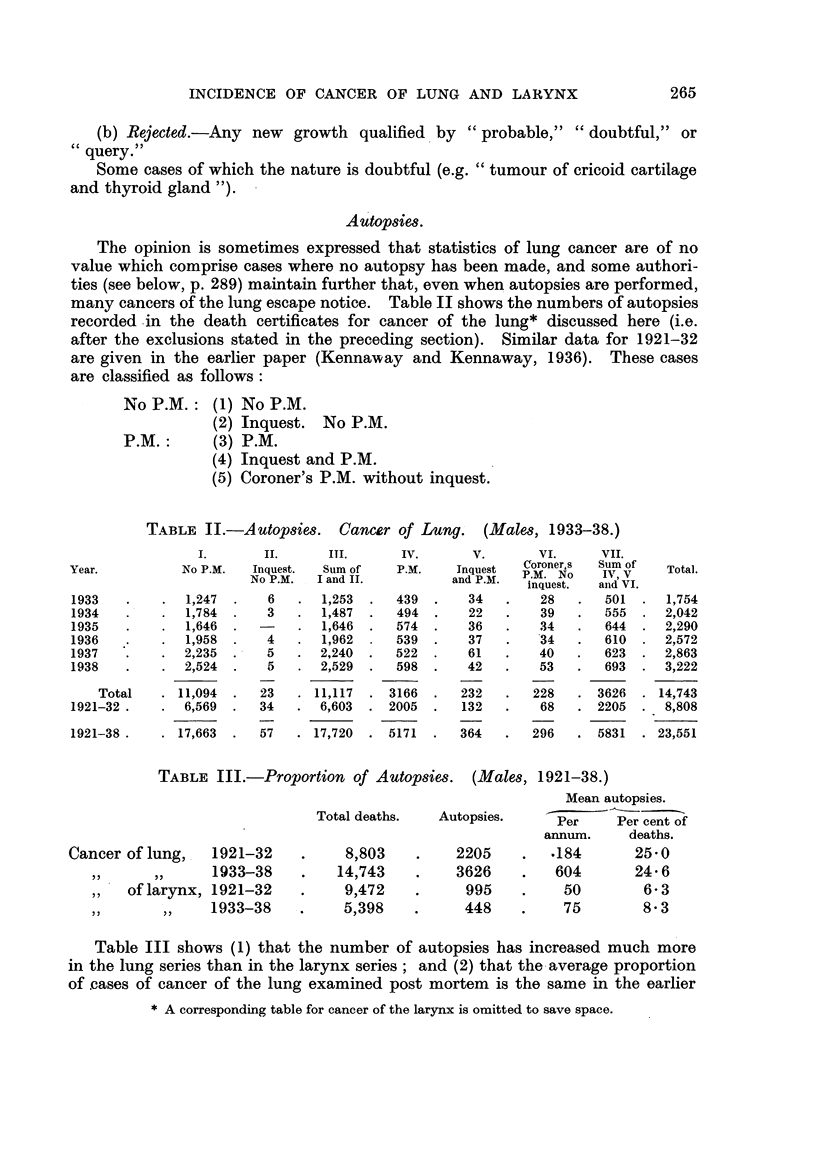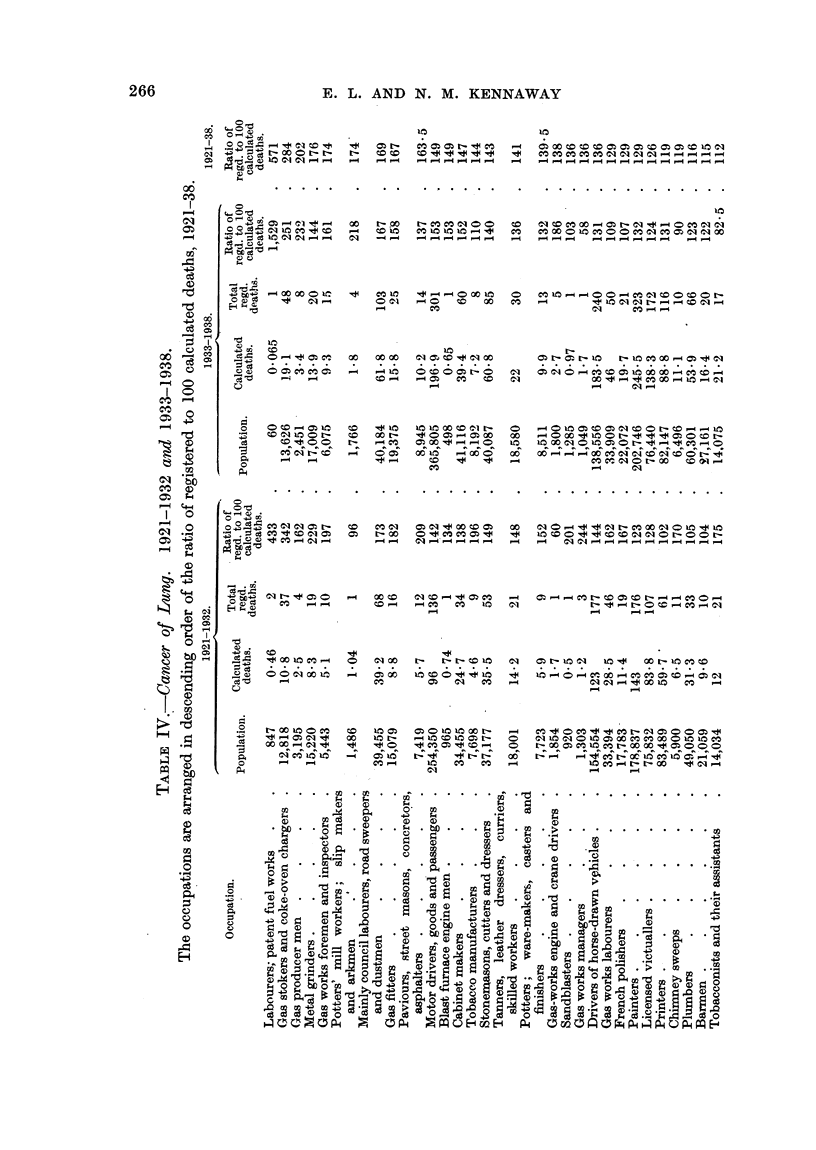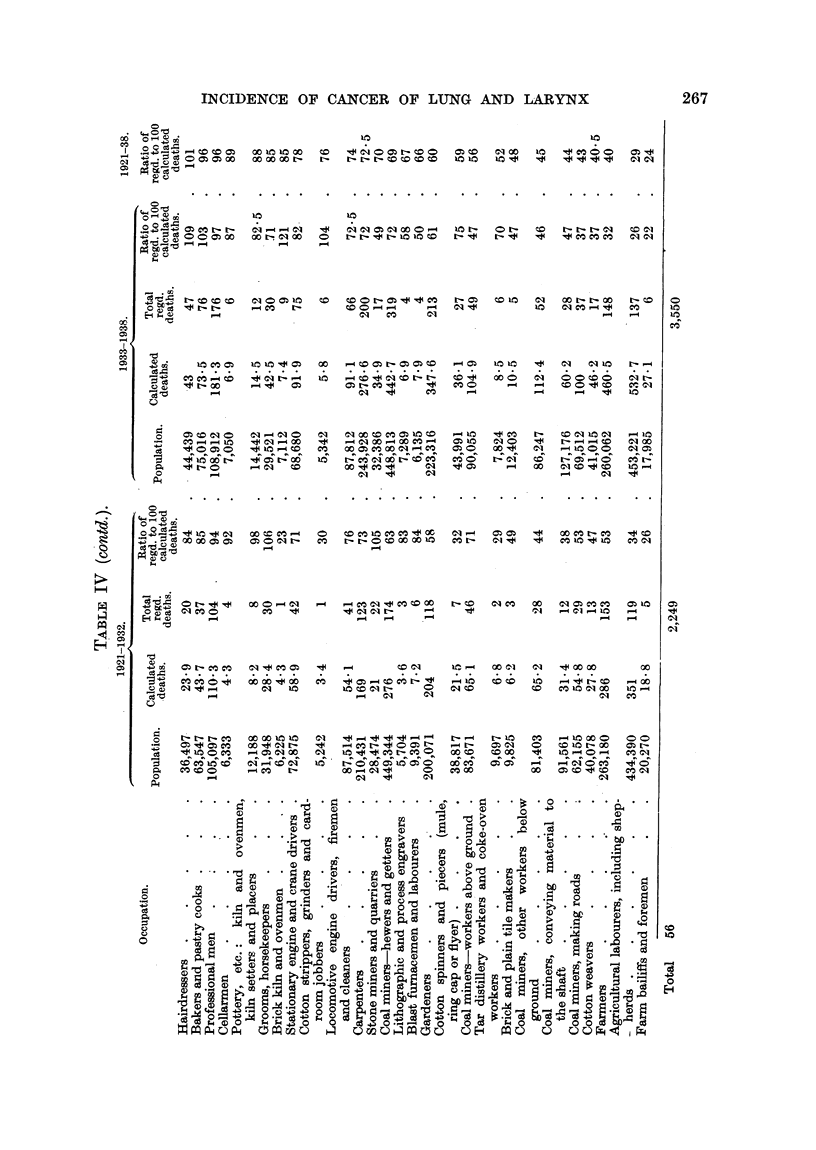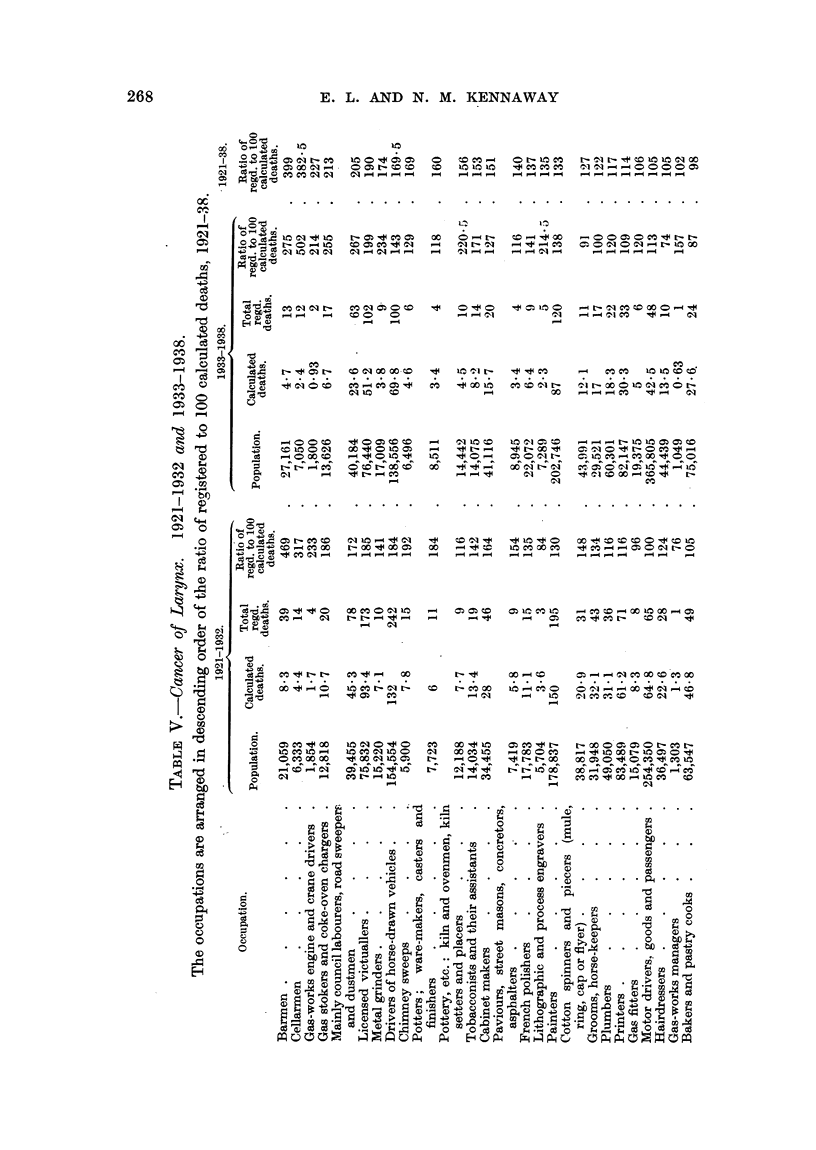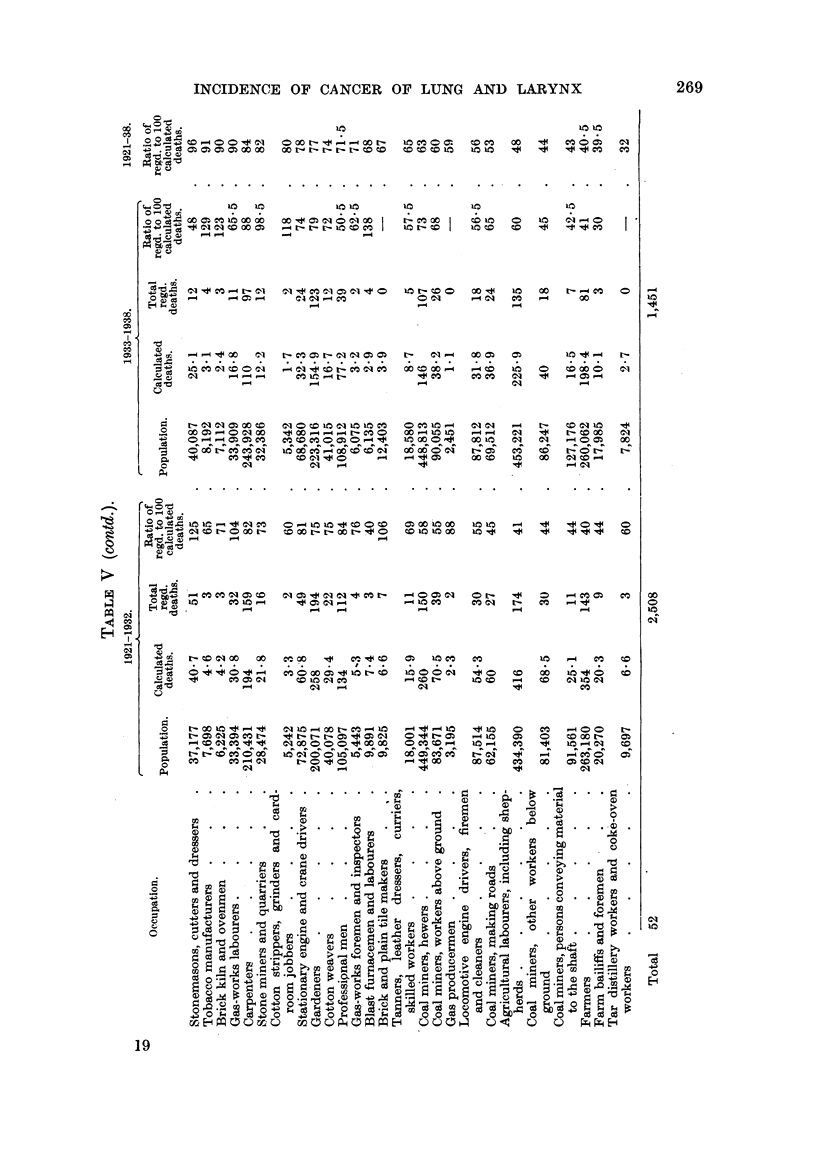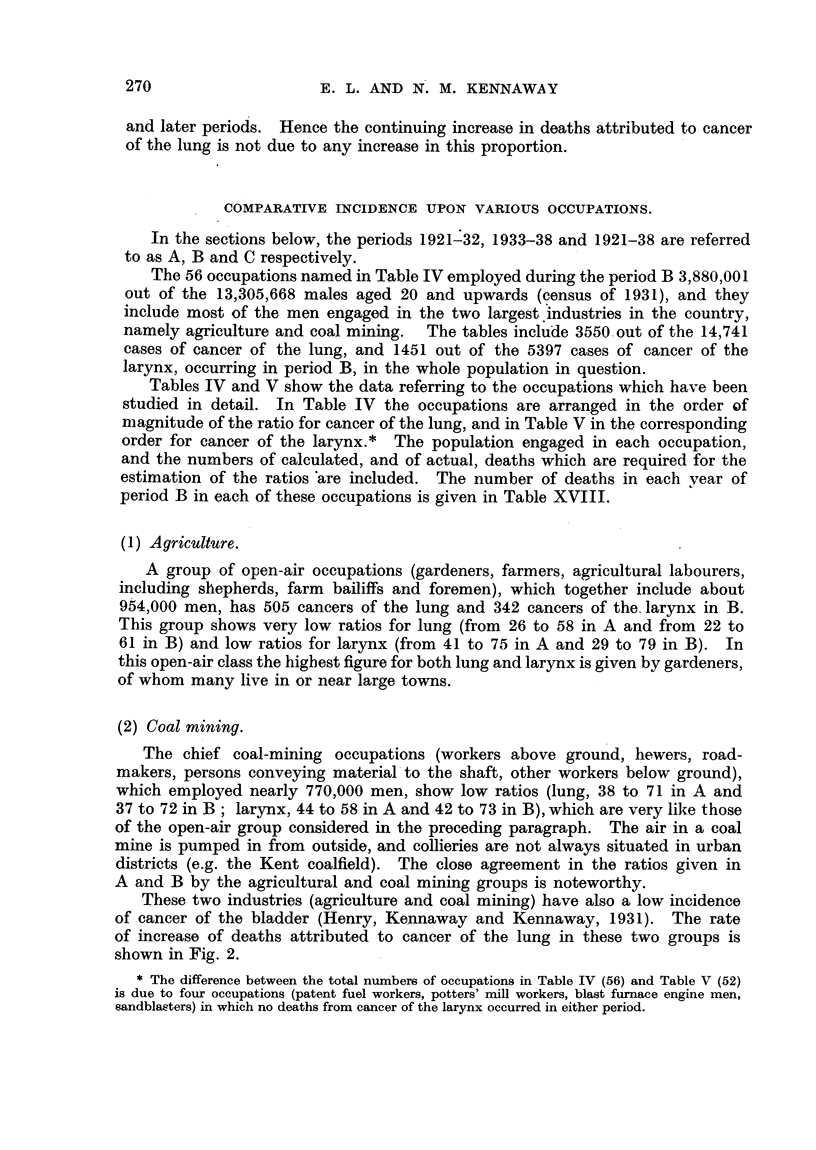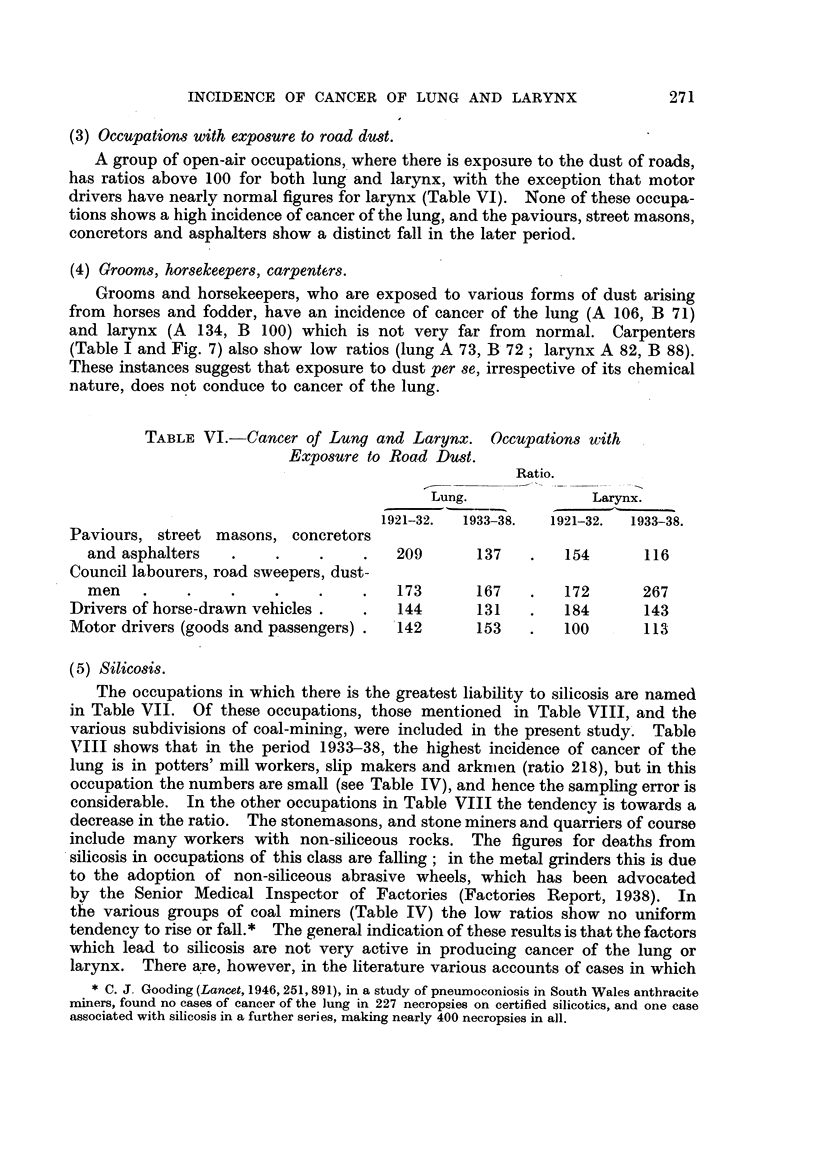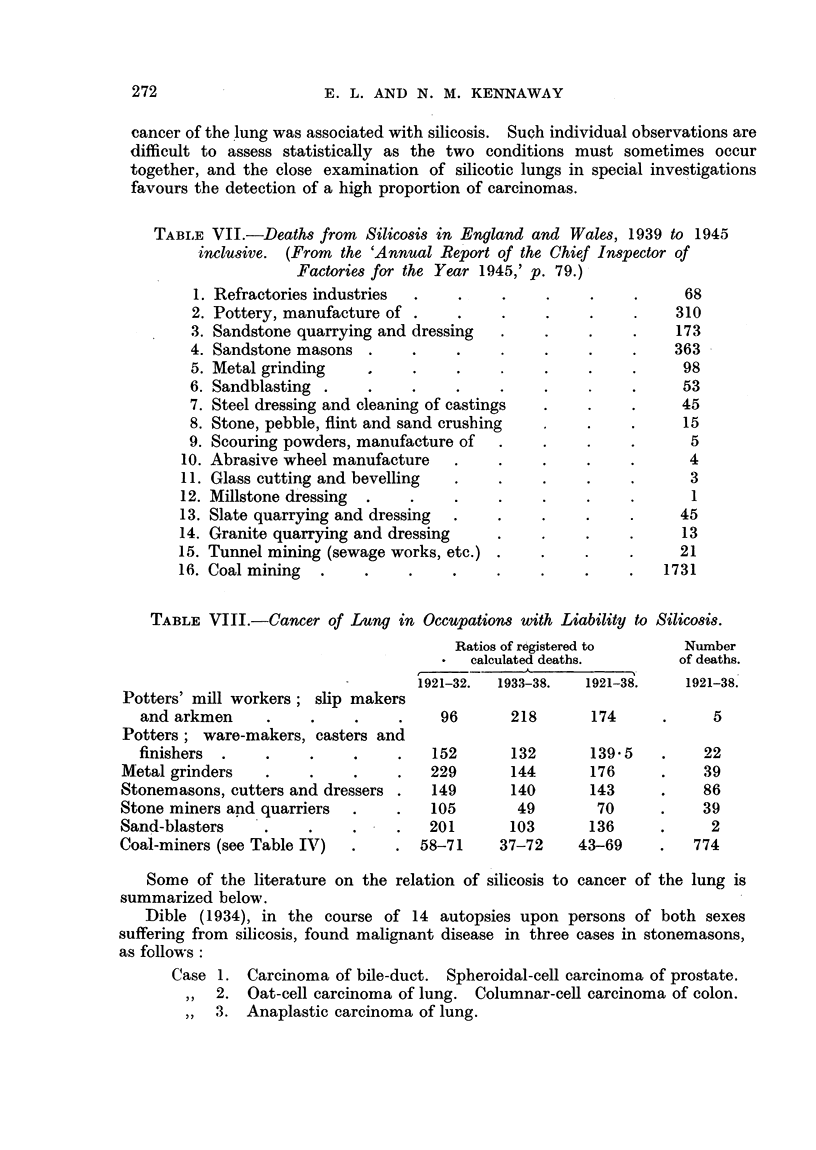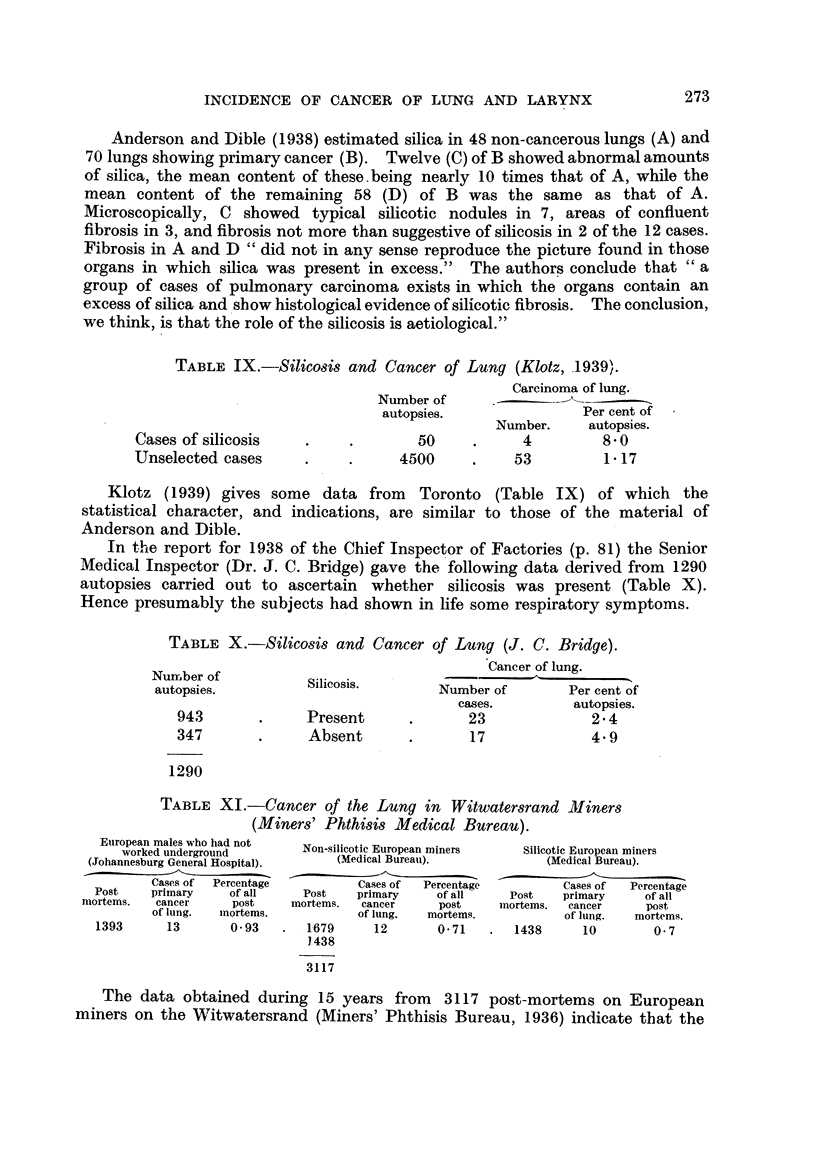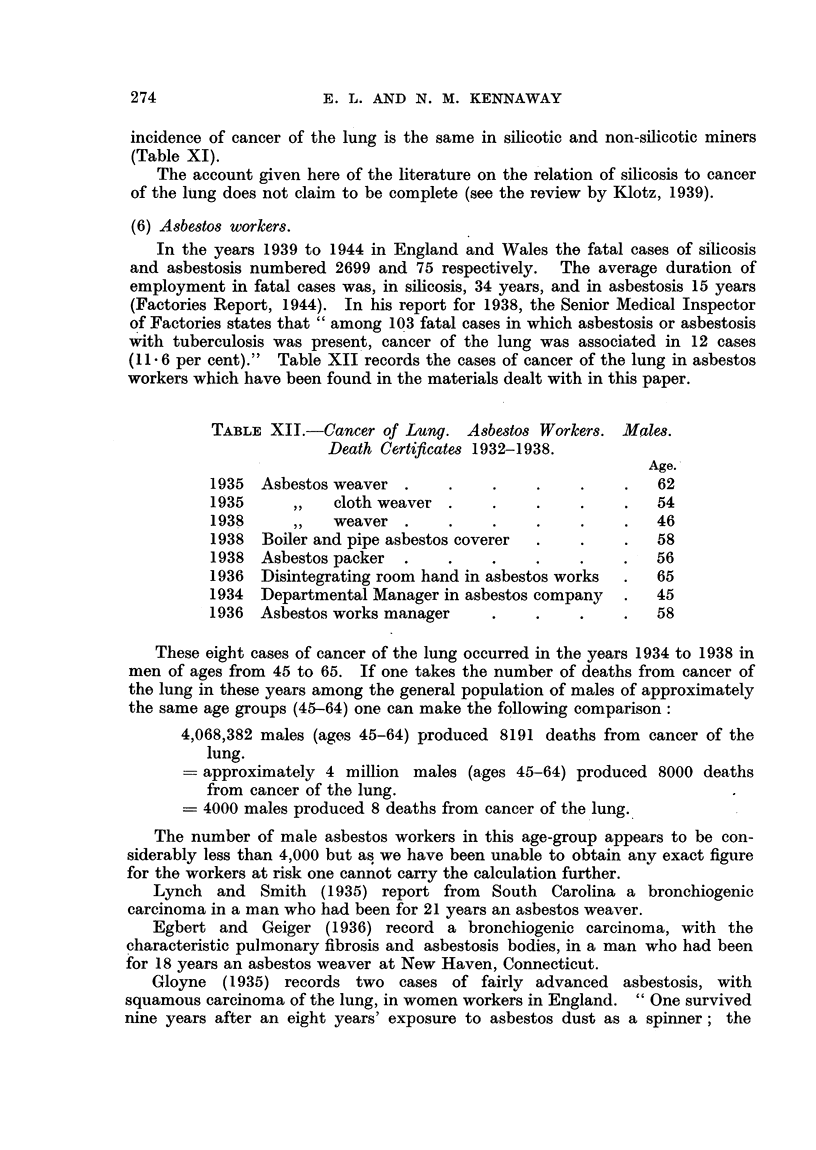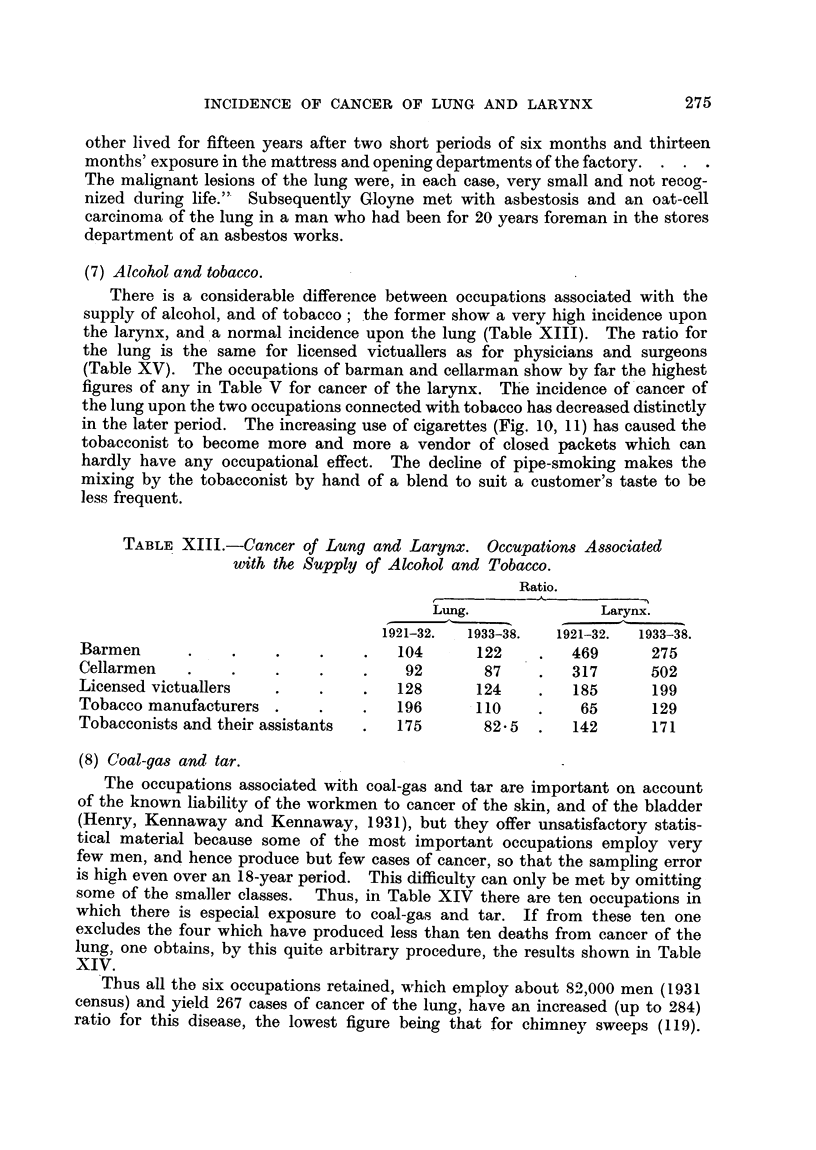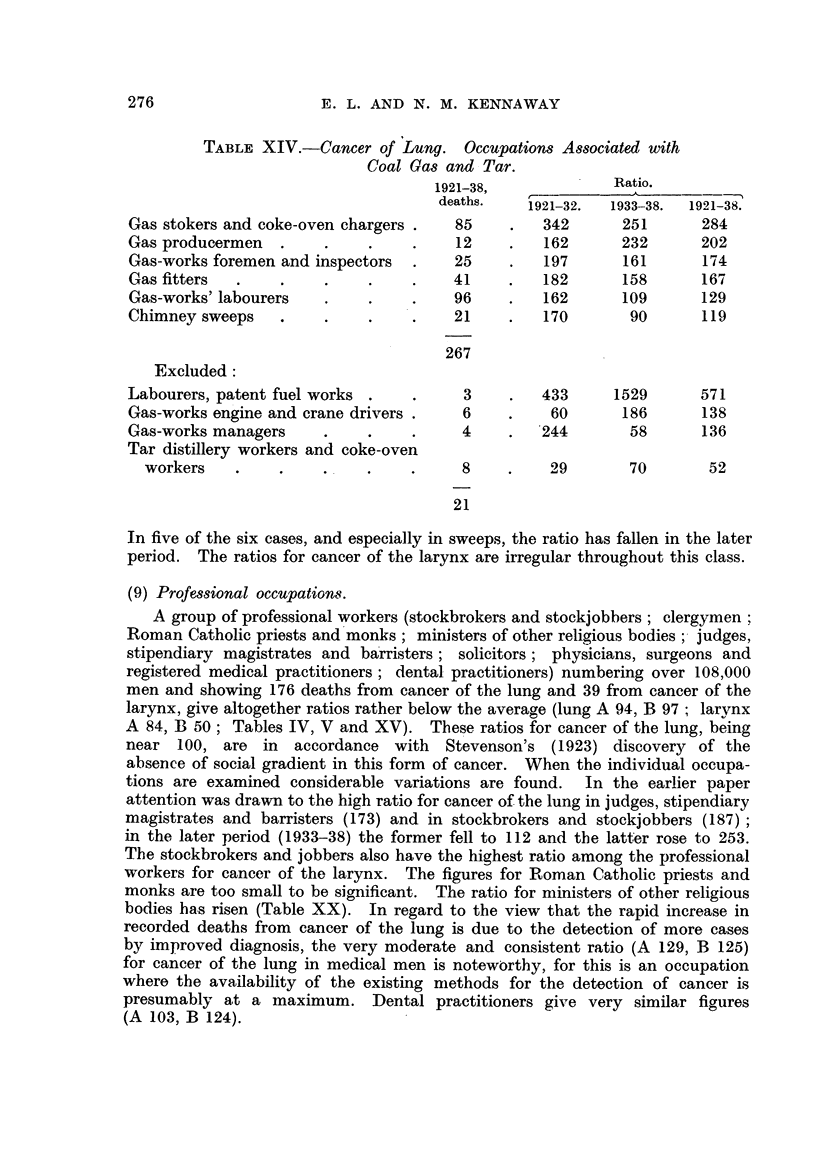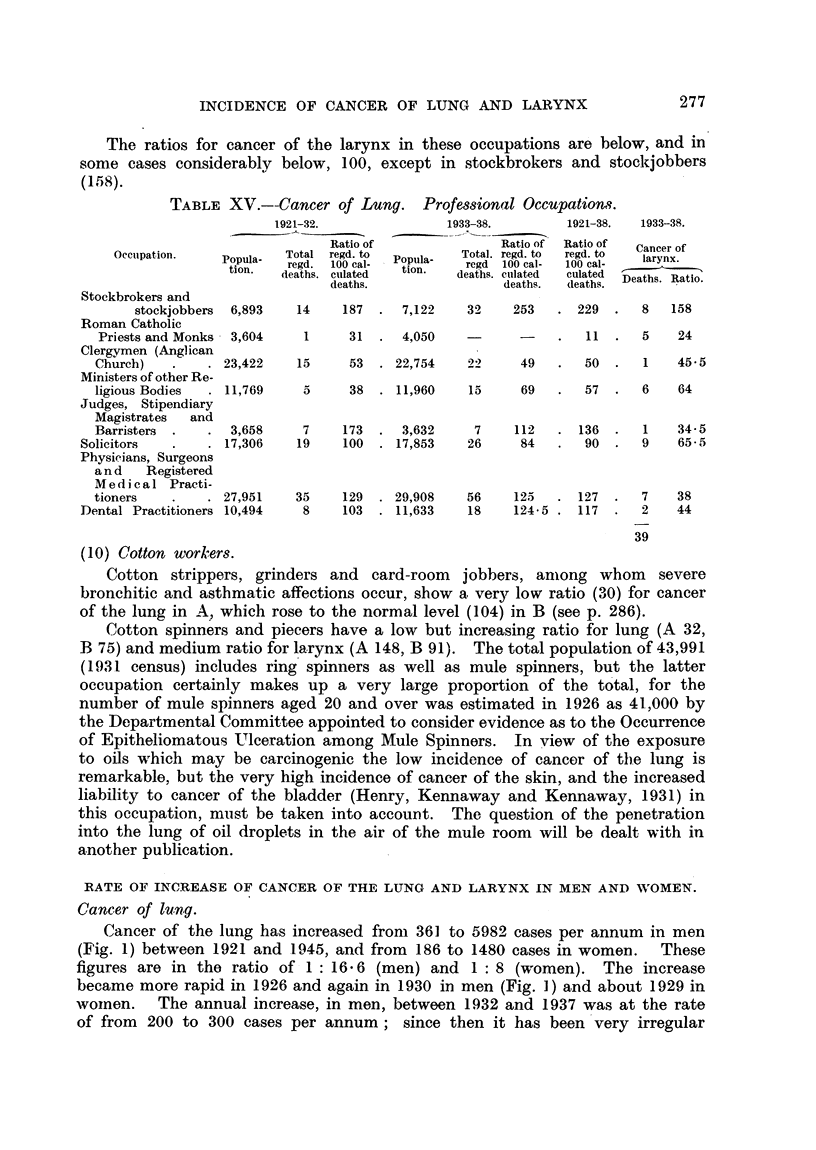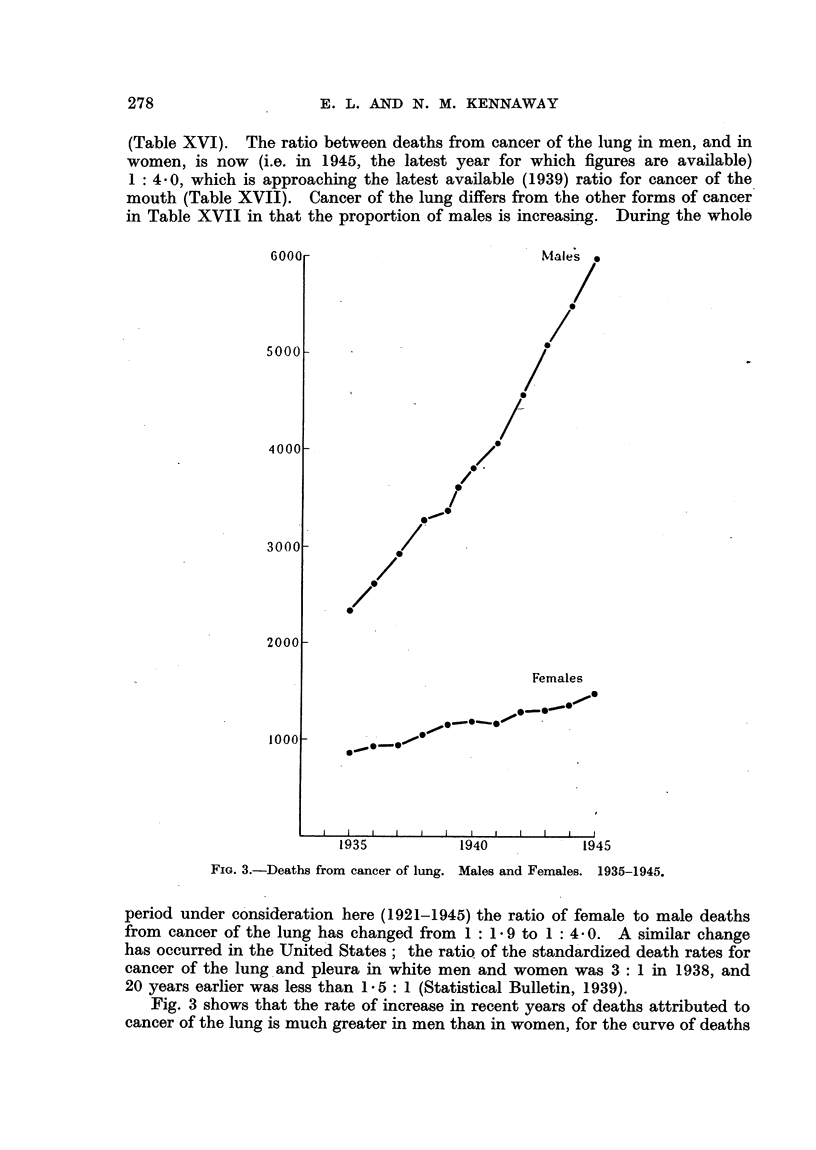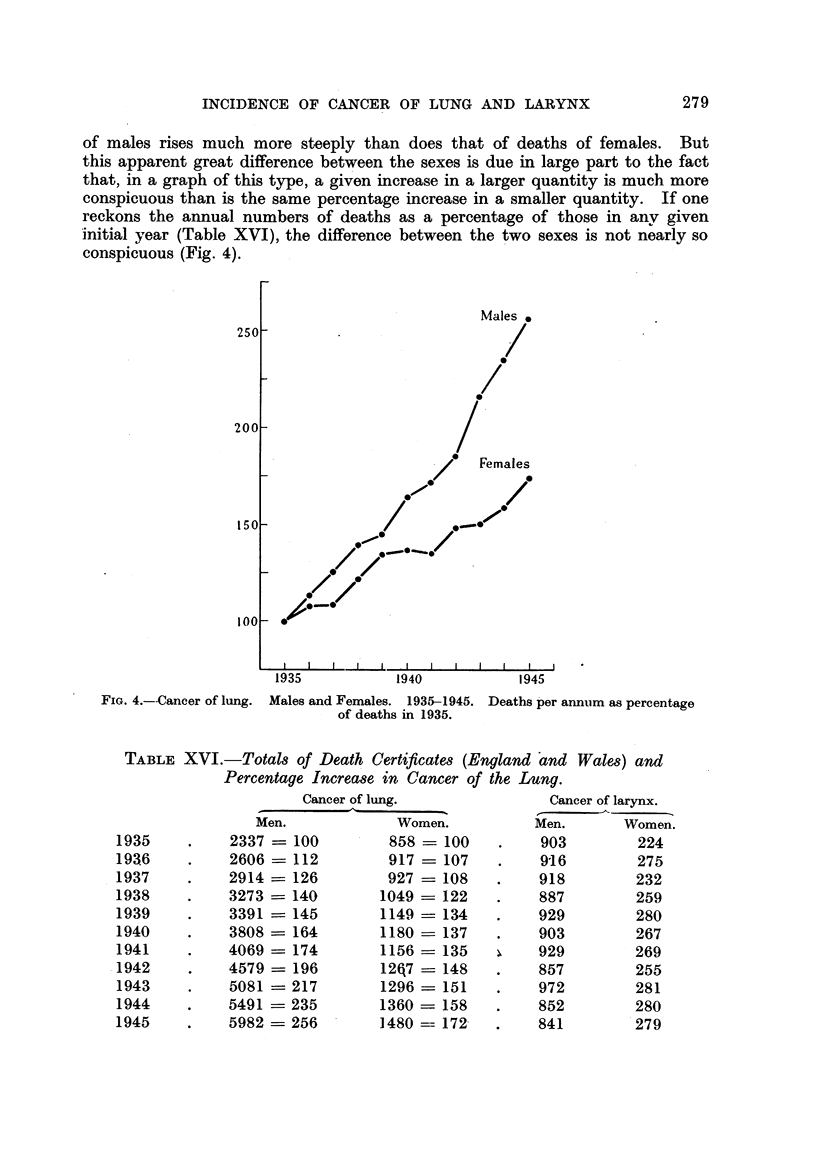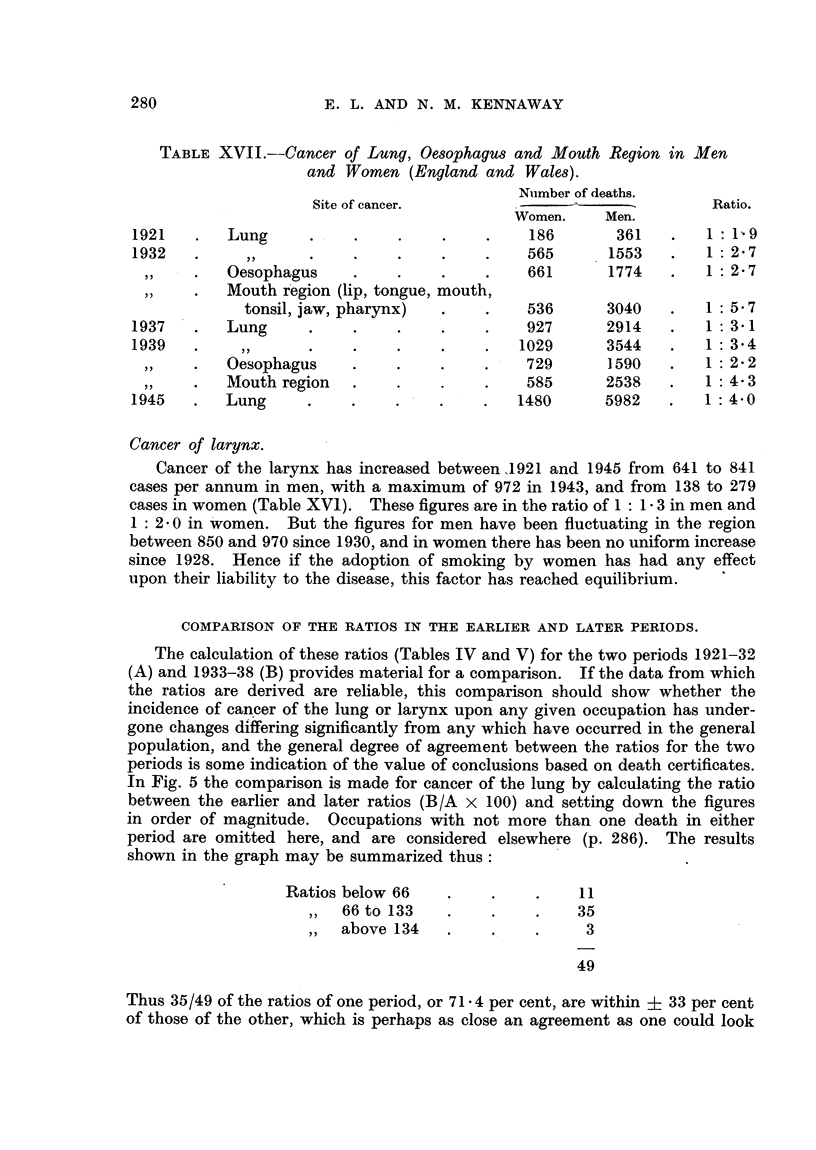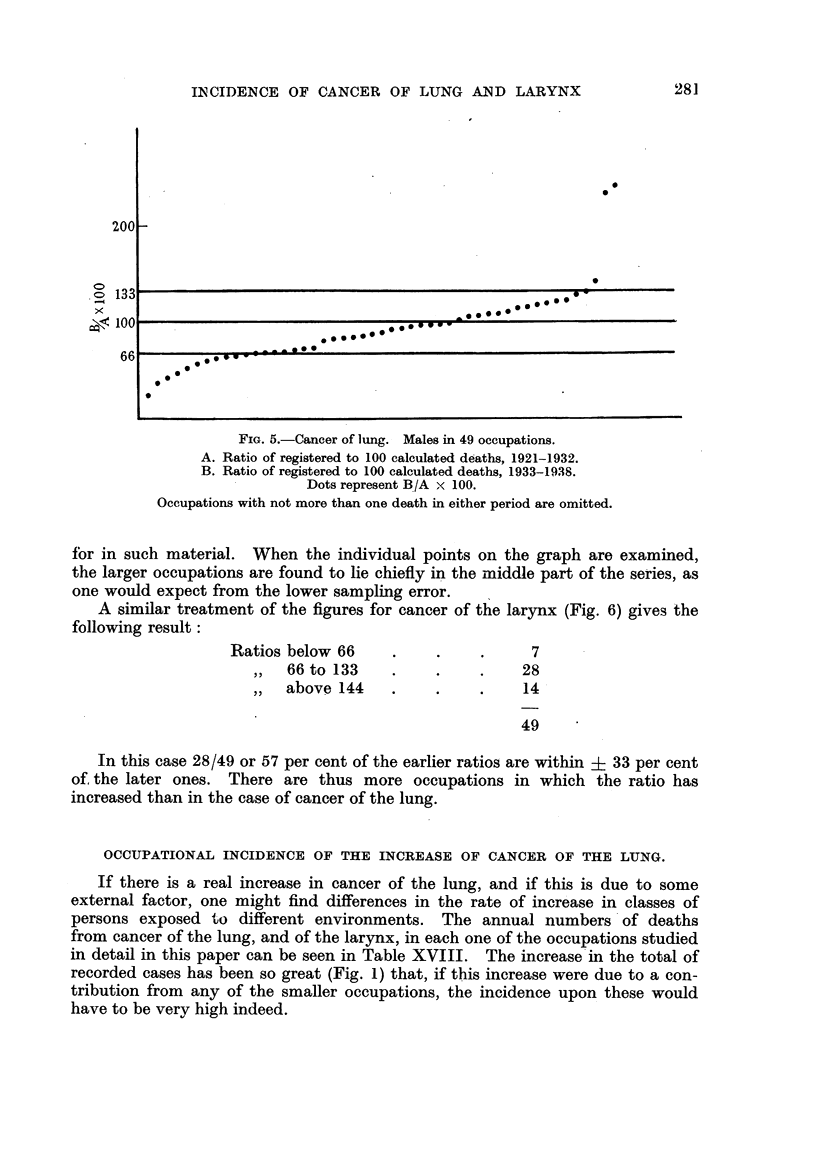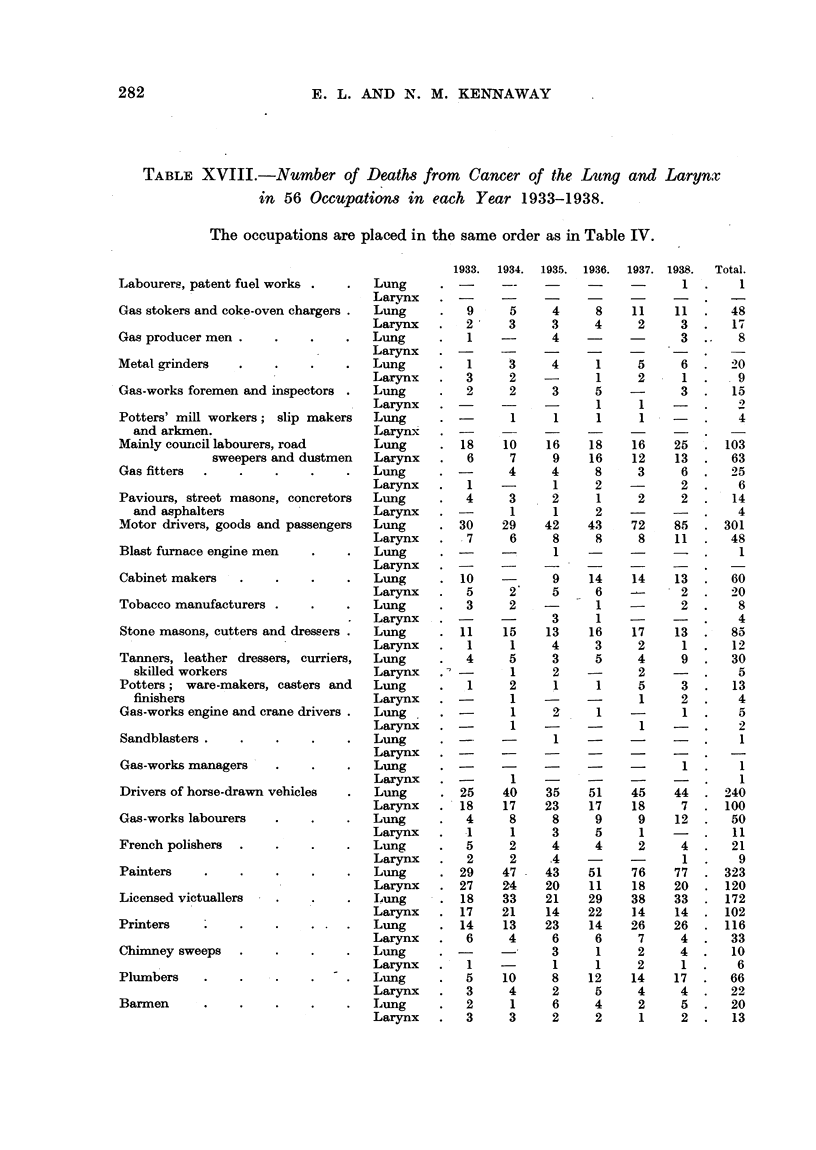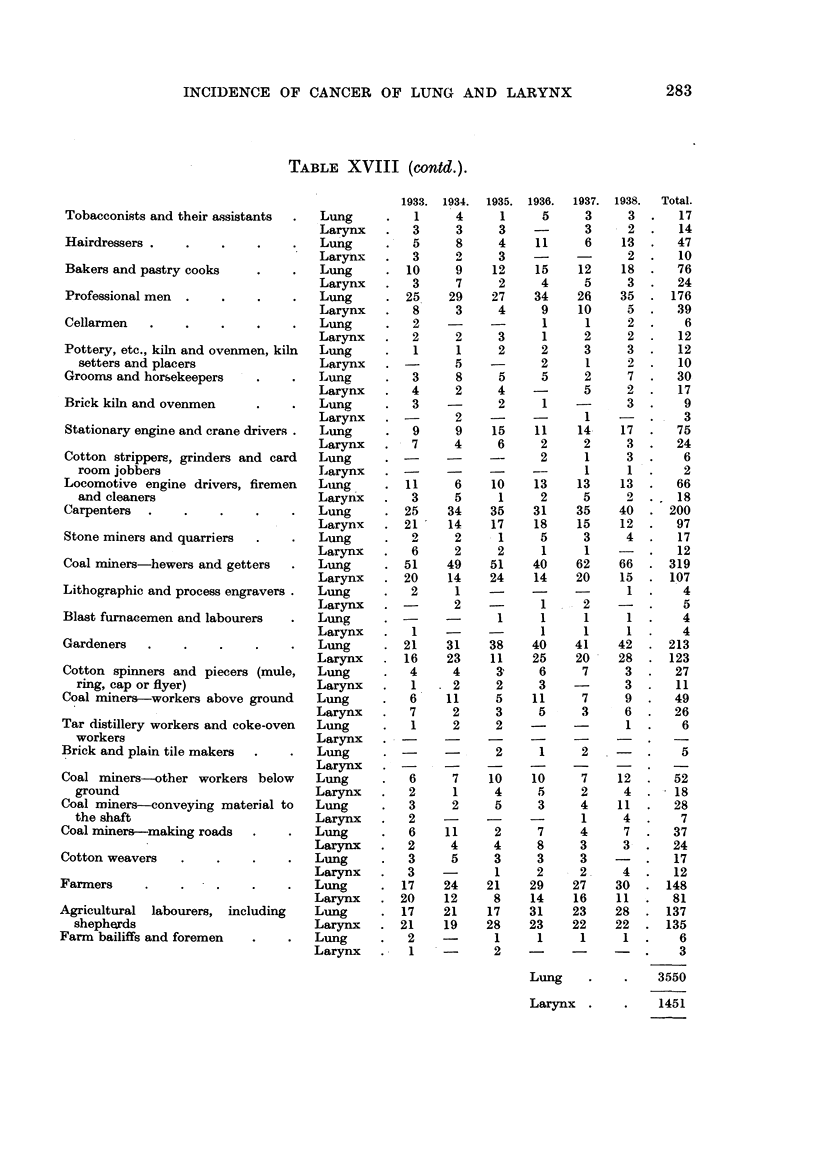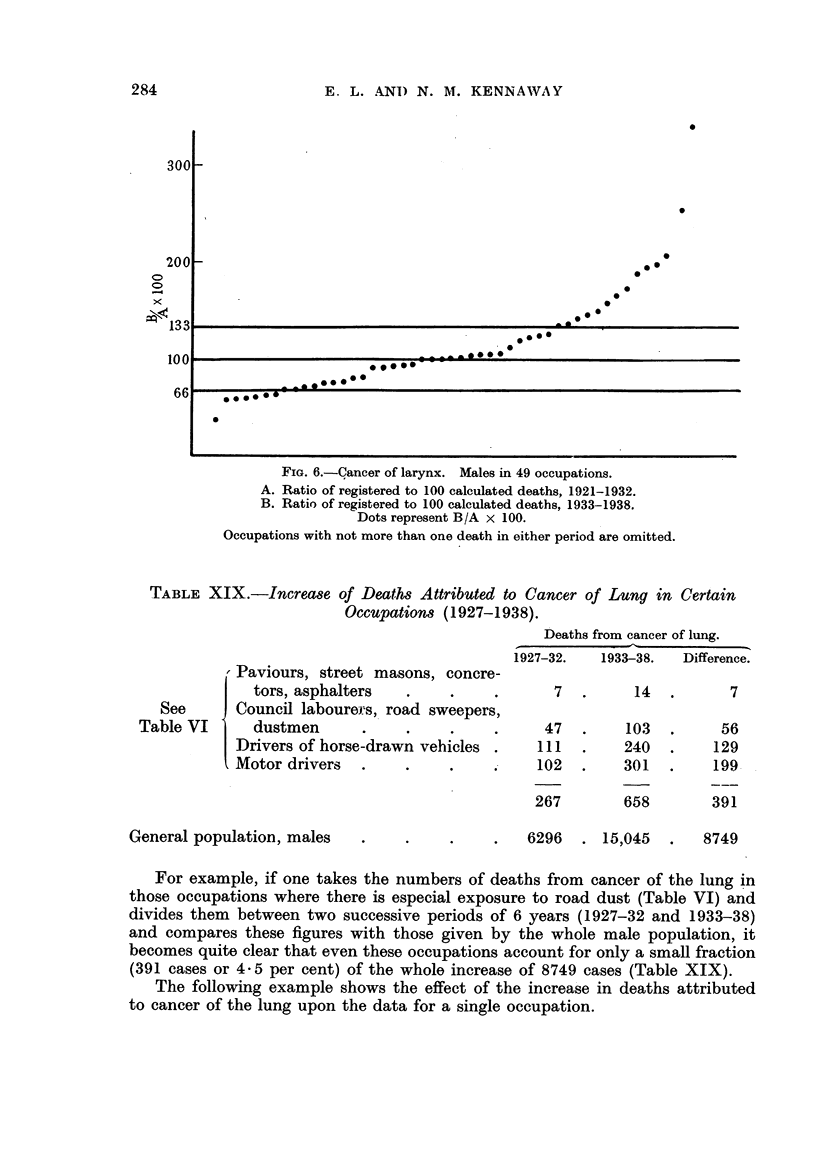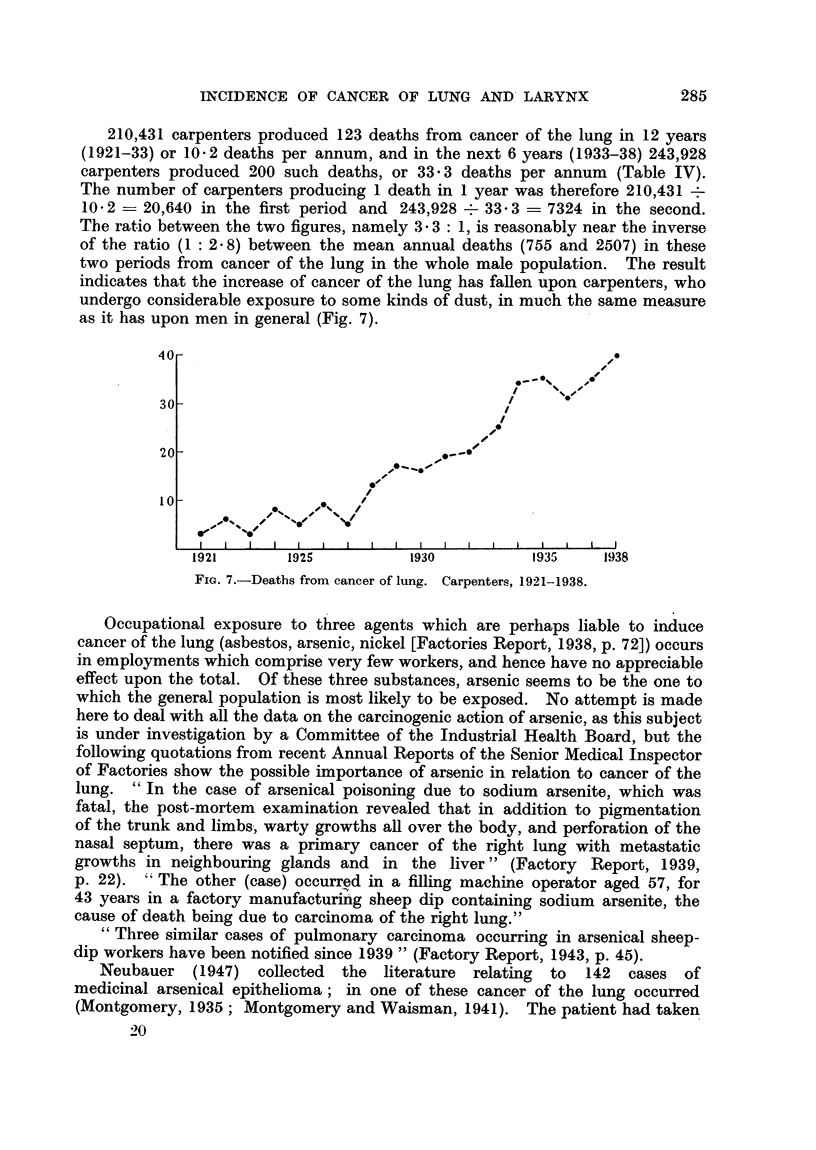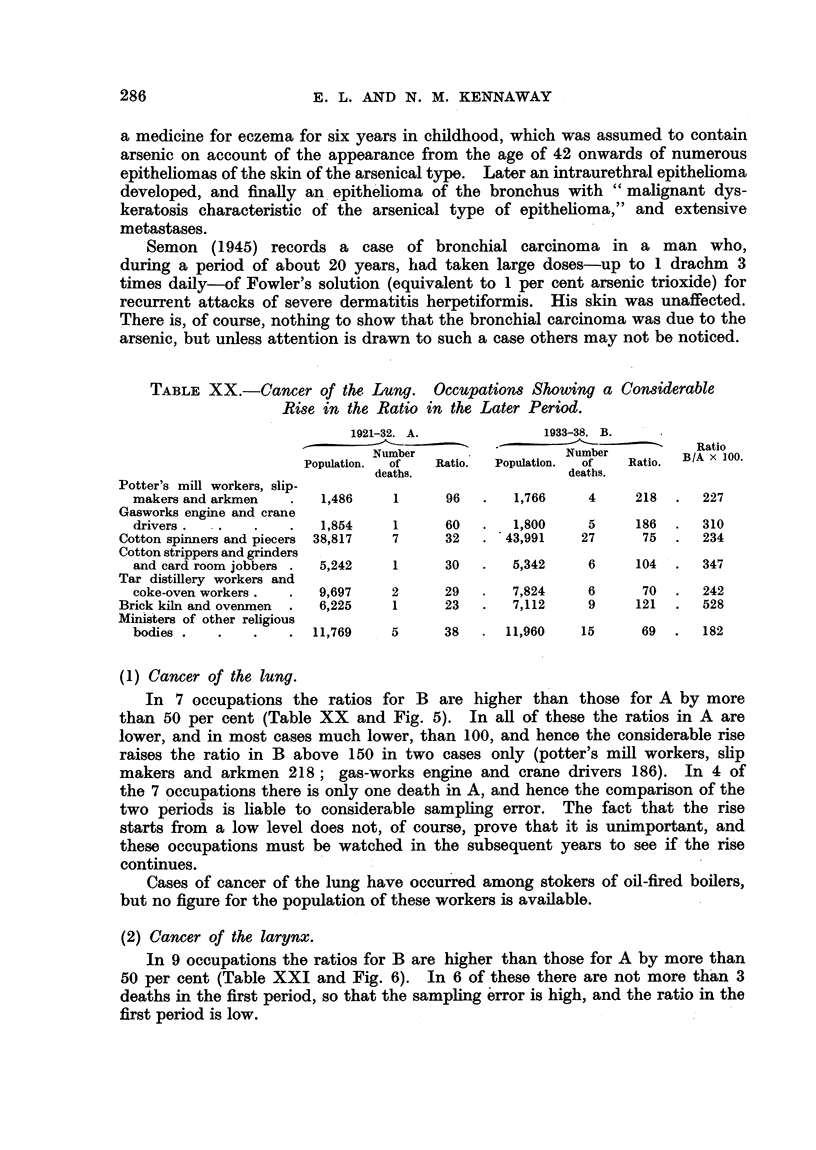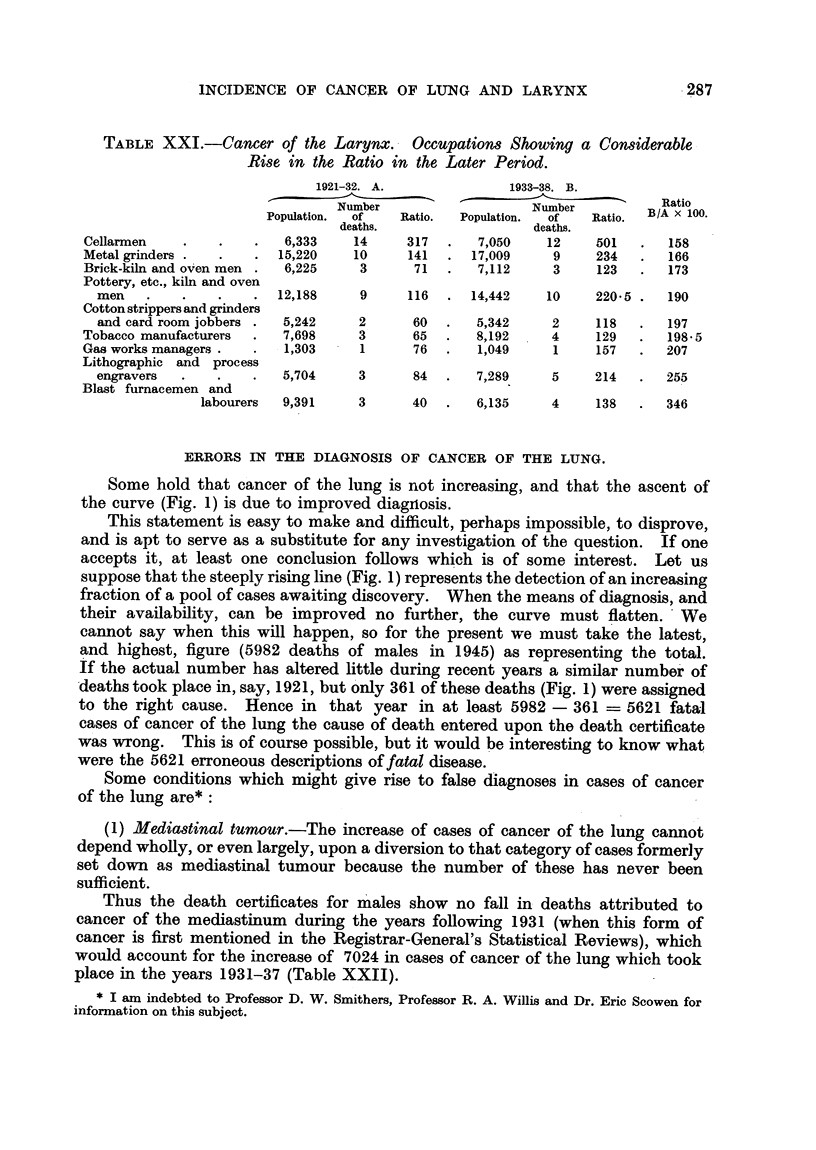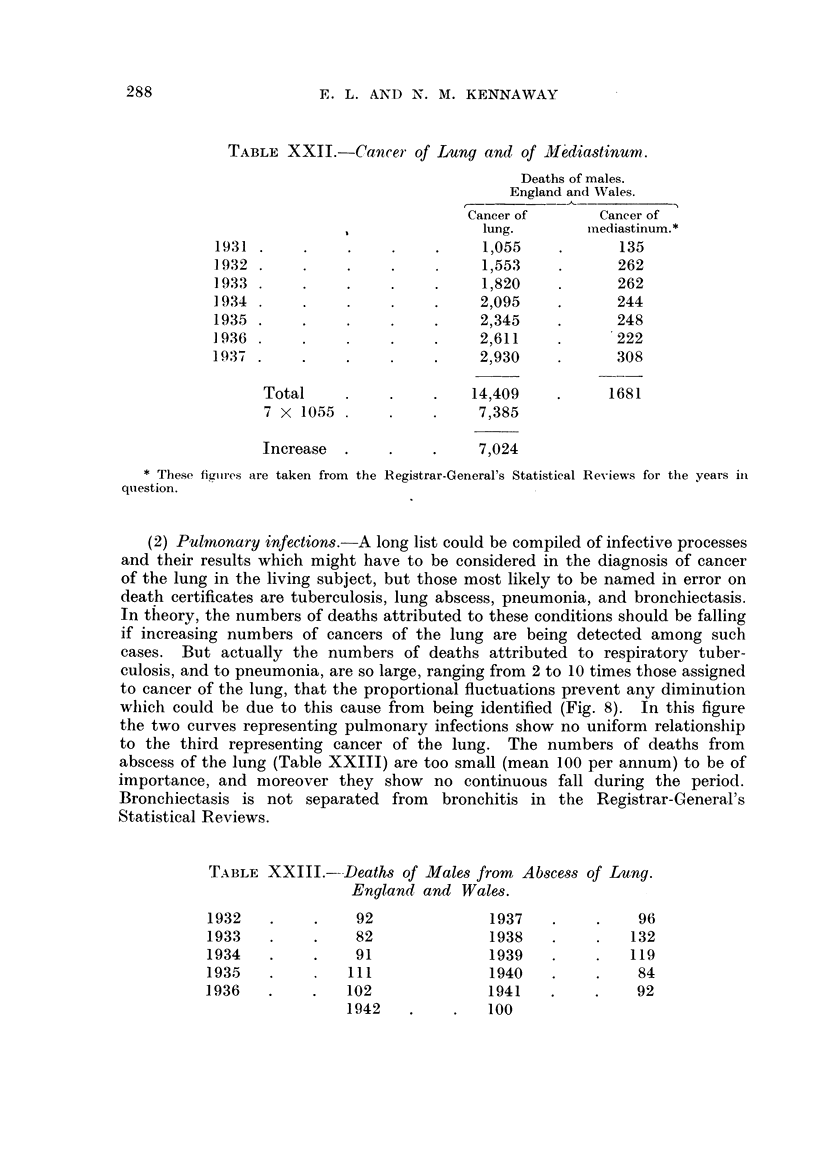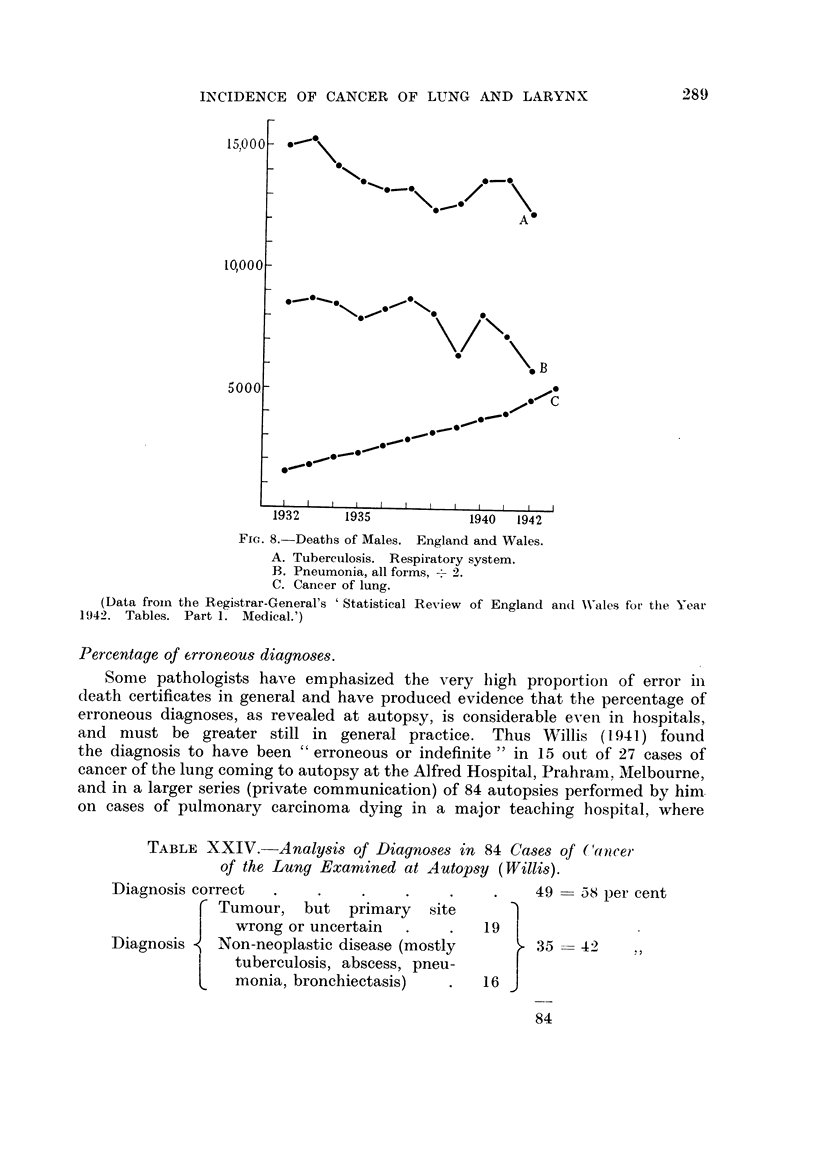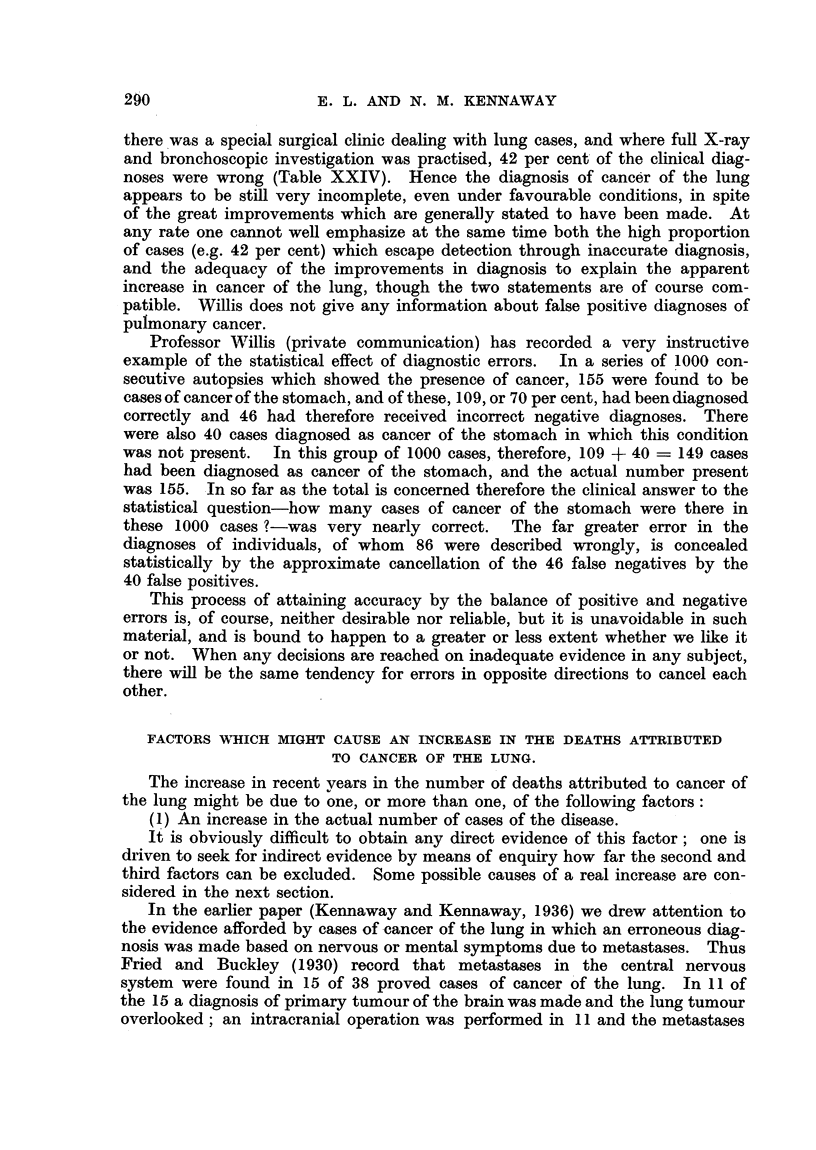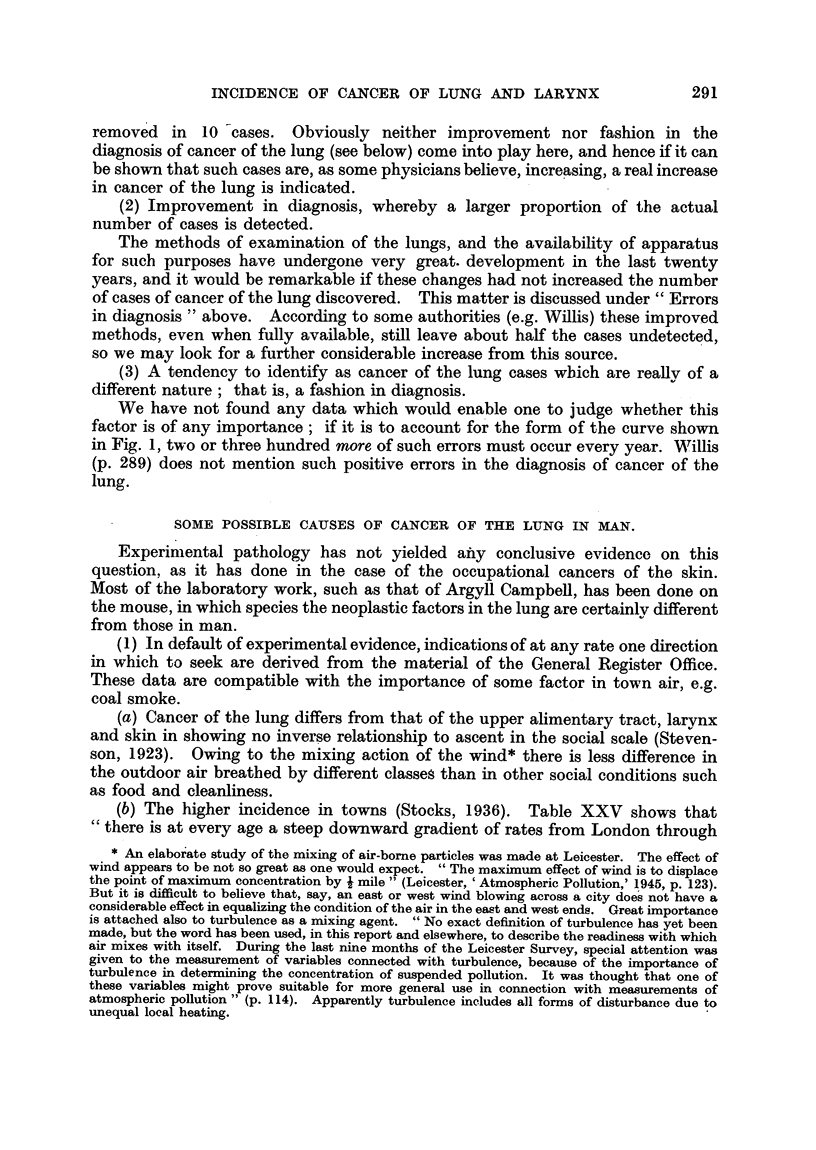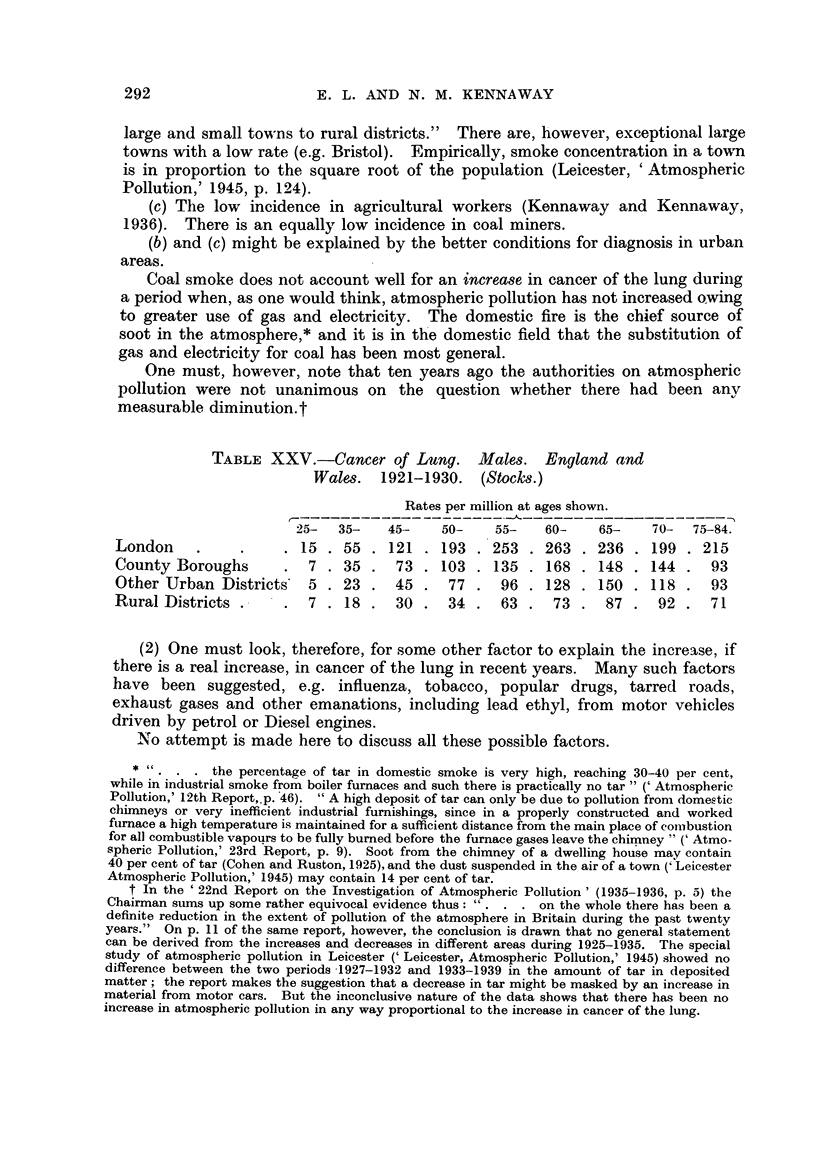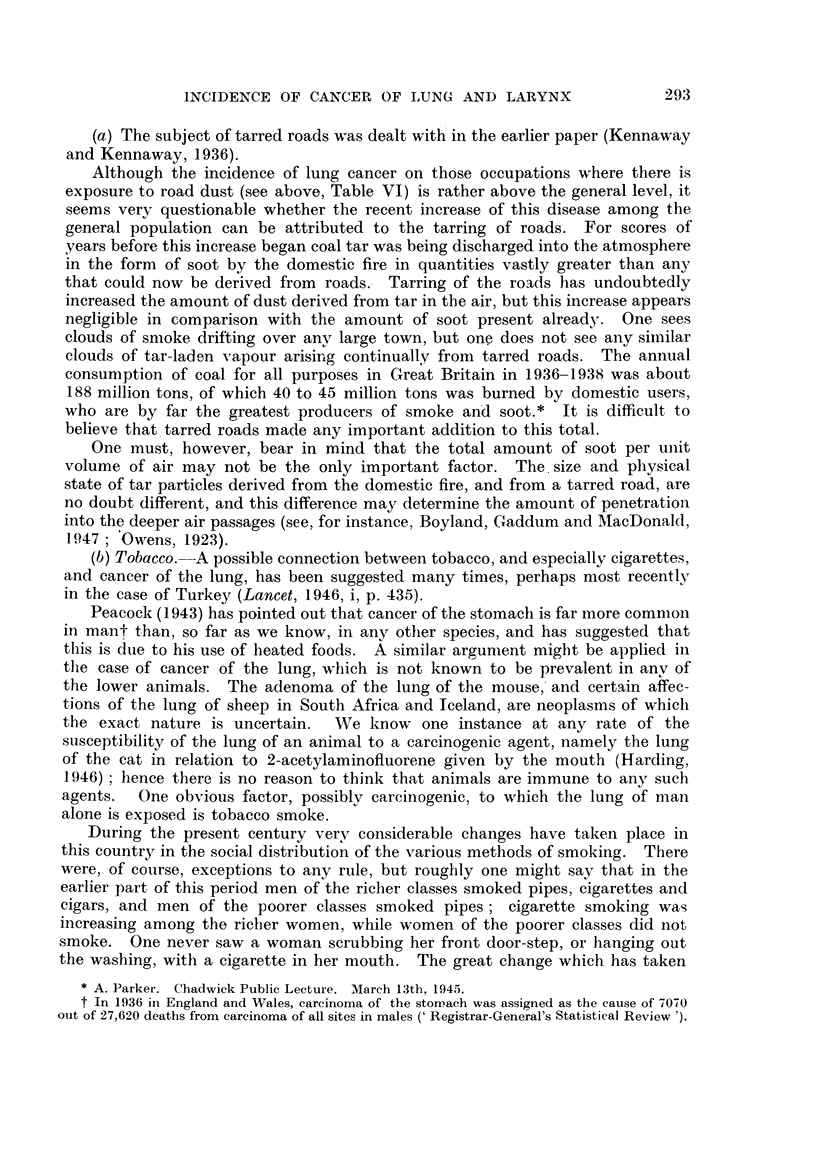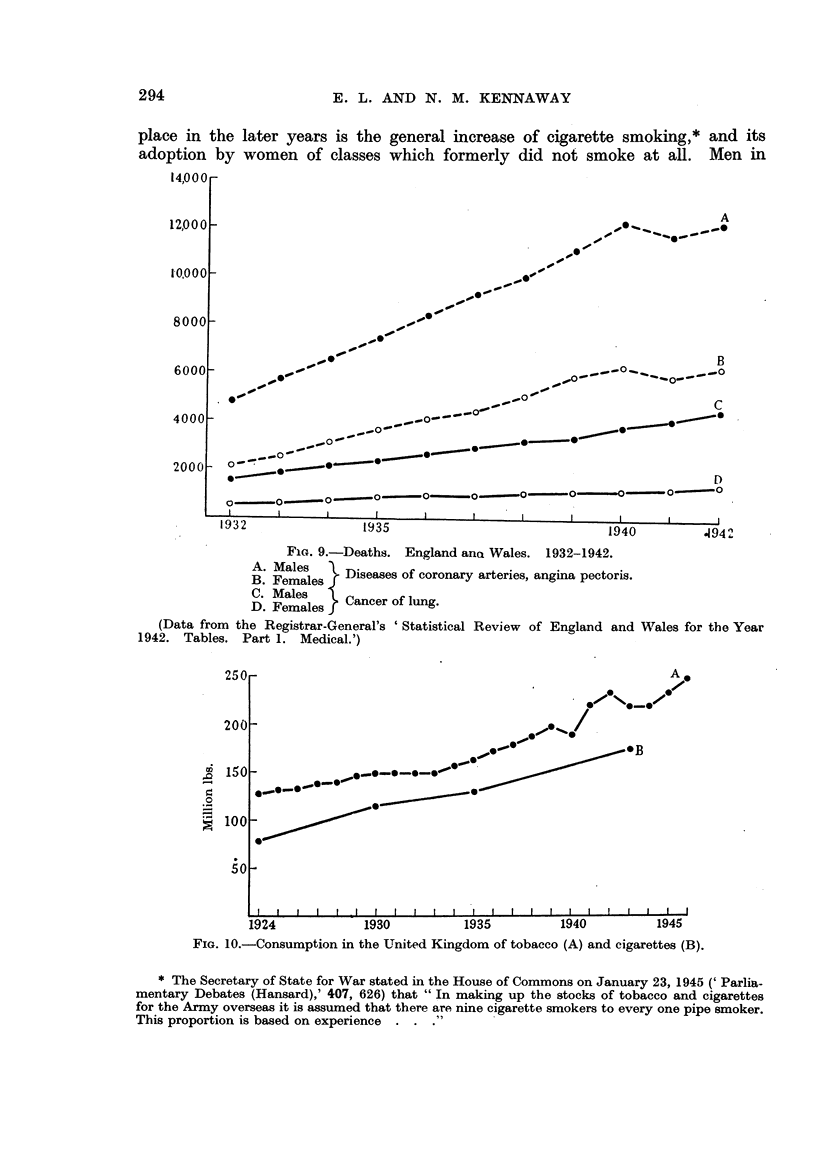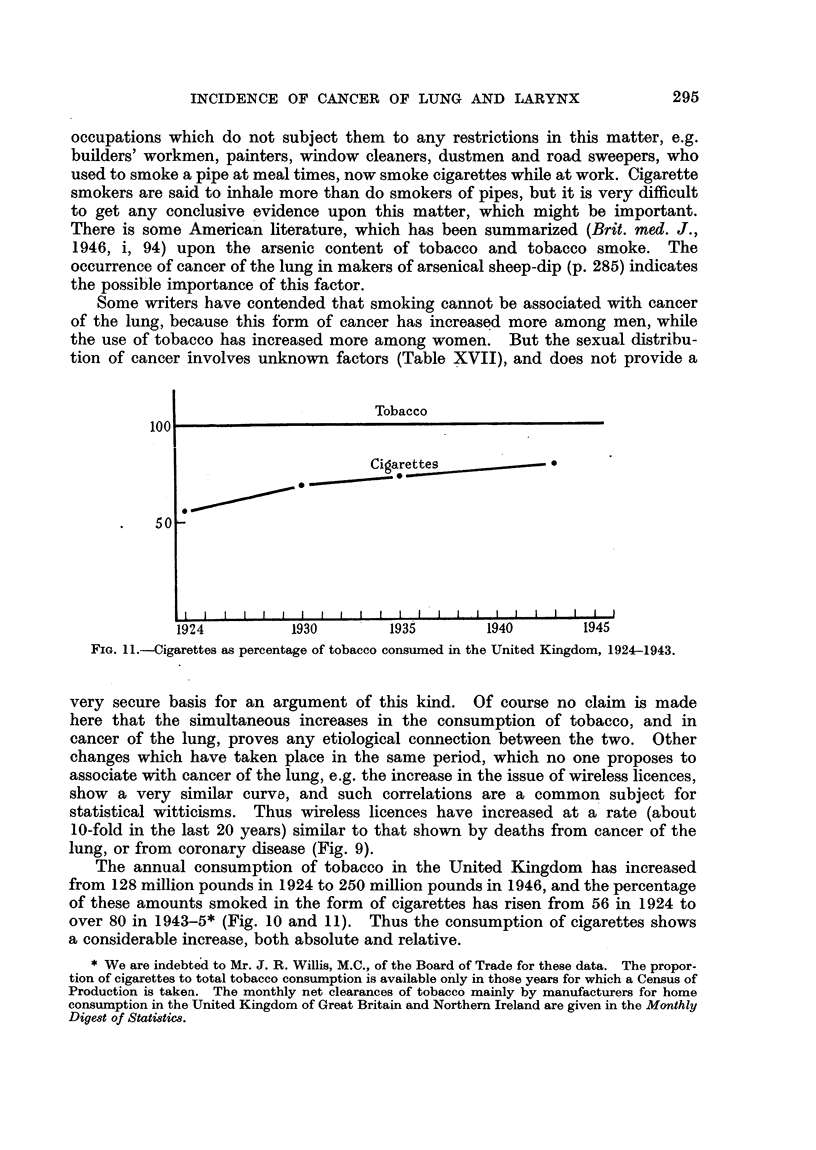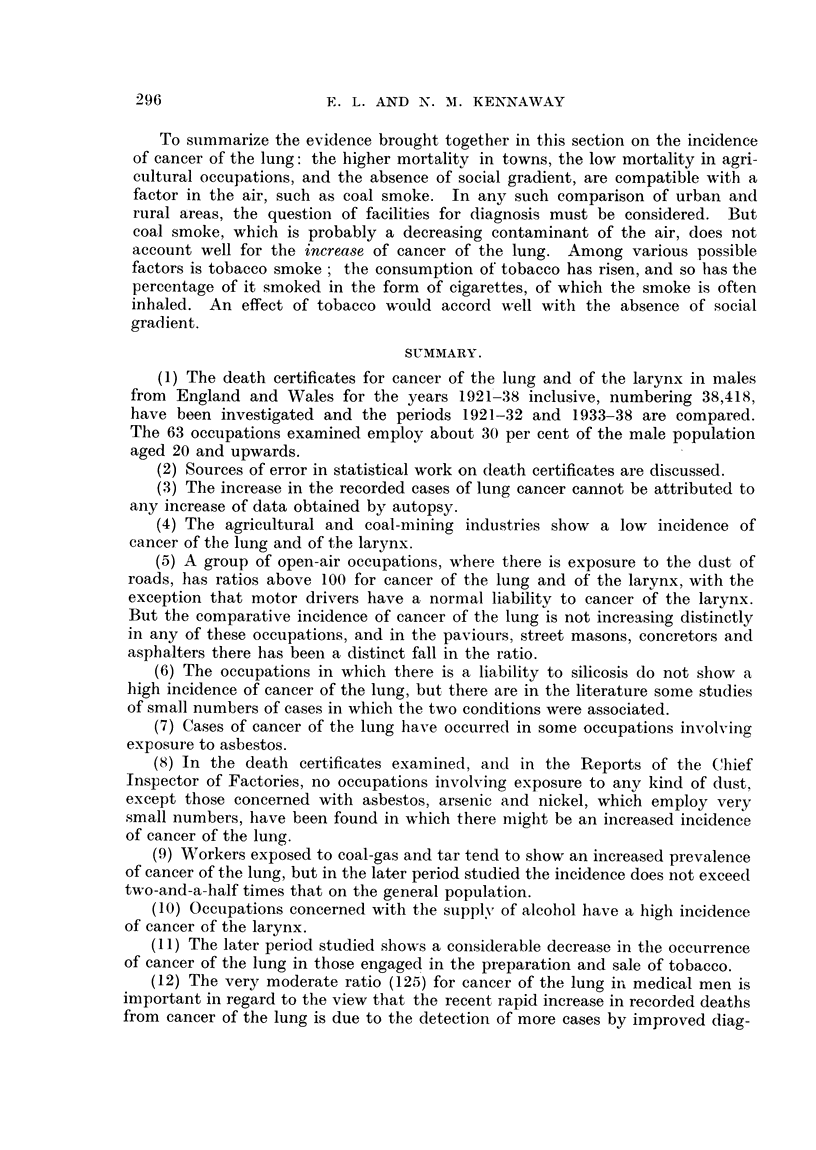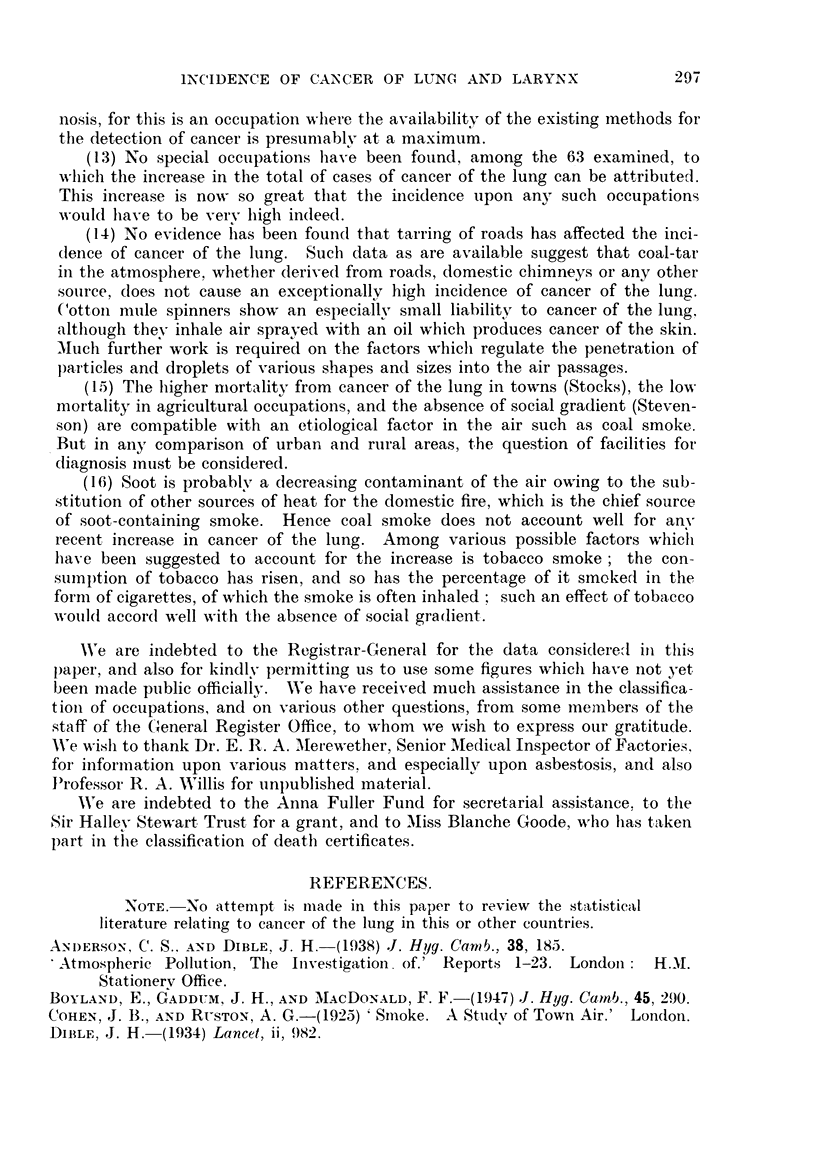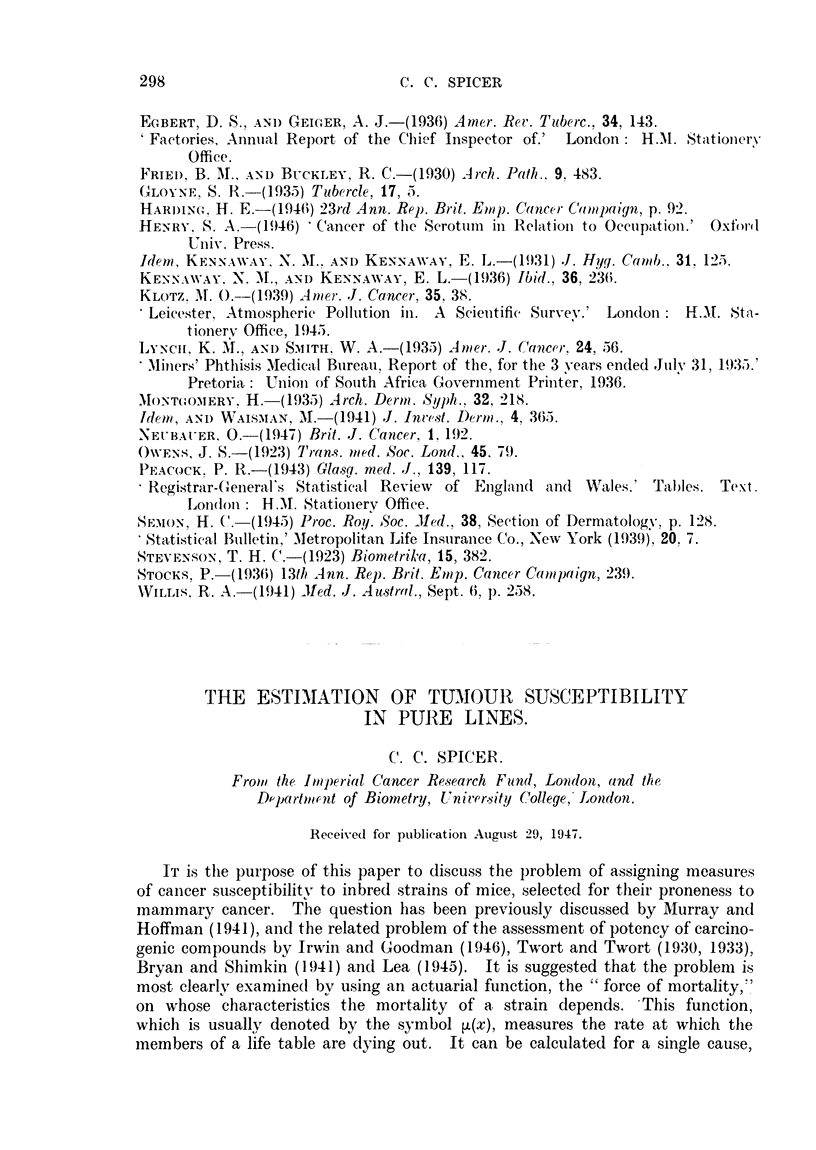# A Further Study of the Incidence of Cancer of the Lung and Larynx

**DOI:** 10.1038/bjc.1947.24

**Published:** 1947-09

**Authors:** E. L. Kennaway, N. M. Kennaway


### A FURTHER STUDY OF THE INCIDENCE OF CANCER OF THE LUNG AND LARYNX.

E. L. AND N. M. KENNAWAY.

(*From the Pathological Department, St. Bartholomew's Hospital, London.*)

Received for publication July 25, 1947.

### MATERIAL EXAMINED.

THE data given below are derived from an examination of the death certificates of cases of cancer of the lung and of the larynx in males from England and Wales for the years 1921 to 1938 inclusive. The figures for the first 12 years of this period (1921 to 1932) were dealt with in an earlier paper (Kennaway and Kennaway, 1936); the material from the subsequent six years has now been examined in the same way, and the data for the whole 18-year period 1921–38 can now be presented together. The whole number of certificates examined individually was 38,418, while the annual total figures for cases in women have been utilized for comparison. This total was made up as follows:

*Certificates Examined.*

| Males. | 1921–32. | 1933–38. | Total. |
|---|---|---|---|
| Cancer of lung | 8808 | 14,741 | 23,549 |
| Cancer of larynx | 9472 | 5,397 | 14,869 |
| | | | 38,418 |

The investigation was carried out on the general lines followed in an earlier study of the occupational incidence of cancer of the bladder and prostate (Henry, Kennaway and Kennaway, 1931).

The chief objects of the enquiry were: (1) to ascertain whether cancer of the lung showed any special incidence upon particular occupations, and (2) to seek for evidence to show whether the increase in deaths attributed to cancer of the lung, now of the order of over 16-fold (from 361 deaths of males in 1921 to 5982 in 1945) indicates any real increase in the prevalence of this form of cancer (Fig. 1). The figures for cancer of the larynx serve as a basis of comparison with those for cancer of the lung, in that the larynx is a part of the same (respiratory) tract which is more accessible to inspection, and is not examined by diagnostic methods which have changed greatly in recent years. The death certificates of persons under 20 years of age were omitted, as the investigation was concerned primarily with the effect of occupation.

### *Method of Calculation of Ratios.*

The comparative incidence of cancer of the lung and larynx upon various occupations was estimated in the manner described in detail in the earlier paper (Kennaway and Kennaway, 1936). In judging of the occupational incidence of any form of cancer it is of course necessary to correct for age distribution, for an occupation employing a large proportion of older men will yield more cases of

FIG. 1.—Deaths from cancer of lung and larynx. Males. 1921–1945.

cancer apart from any aetiological factor. The census returns give the age distribution, in 5- and 10-yearly periods, of the whole population of males, and also of those following each one of the recognized occupations, at the time of the census. The number of deaths attributed to cancer of the lung and larynx occurring in the whole male population in each of these age groups during the years in question was obtained from material at the General Register Office. The comparison of the various occupations with the general population is then a sum in proportion, which gives the ratio of the number of deaths in any given occupation to those, reckoned as 100, which occurred in the same number of males in the same age groups in the whole population. An example of this calculation is given in the earlier paper.

### *Sources of Error in Statistical Work on Death Certificates.*

These were examined in the earlier paper (Kennaway and Kennaway, 1936). The use of death certificates for statistical purposes is liable to errors which are inherent in the nature of the material and cannot be wholly eliminated. One must either recognize and admit these errors and correct them in any way possible, or abandon any attempt to obtain information from these data.

(1) *Correction for period between census.*—In order to compare the incidence of cancer upon different occupations one must know the number of persons following each occupation. This is learned from the census returns. The longer the inter-census interval the greater is the risk of important alteration in these numbers. The figures for populations used for the earlier period (1921–32) were those of the 1921 and the 1931 census, which were combined by means of a formula described in the earlier paper (Kennaway and Kennaway, 1936) ; those in the present paper are from the 1931 census, which is the last taken. No census was made in 1941, on account of the war.

The ratios for the whole 18-year period are calculated from the sums of the registered, and the calculated, deaths for the 12-year and 6-year periods as in the following examples :

TABLE I.—*Combination of 12-year and 6-year Periods. Cancer of the Lung.*

| | 1921–1932. | | | 1933–1938. | | | 1921–1932 + 1933–1938. | | |
|---|---|---|---|---|---|---|---|---|---|
| | Calculated deaths. | Total registered deaths. | Ratio of regd. to 100 calculated deaths. | Calculated deaths. | Total registered deaths. | Ratio of regd. to 100 calculated deaths. | Calculated deaths. | Total registered deaths. | Ratio of regd. to 100 calculated deaths. |
| Carpenters | 169 | 123 | 73 | 276·6 | 200 | 72 | 445·6 | 323 | 72·5 |
| Printers | 59·7 | 61 | 102 | 88·8 | 116 | 131 | 148·5 | 177 | 119 |

(2) *Sampling error.*—In some of the most interesting occupations from the present standpoint (e.g. patent fuel workers, tar distillers) a few thousand men only are employed, and these will yield but very few cases of cancer of any one organ even if the incidence is high (Table XIV). Hence the sampling error will be large even over the period studied here of 18 years. The figures given in Table XVIII show the range of fluctuations which occurs. Even the large group of agricultural workers, numbering over 900,000 men, does not give so smooth a curve representing deaths from cancer of the lung as does the whole male population (Fig. 2).

Factors which may affect the value of the individual death certificate are :

(3) *Duration of employment.*—The individual entry in the death register does not give, and is not intended to give, any information about the length of

FIG. 2.—Deaths from cancer of lung. Males.
A. General population. 1921–1944.
B. Coal miners.
C. Agricultural workers. } × 20. 1921–1938.

time during which the deceased followed the occupation named, nor about the nature or duration of any previous employment ; the uncertainty could be removed only by enquiry into each individual case, and that is impracticable when large numbers are concerned. The immense amount of labour involved in such enquiries can be seen in the monograph on cancer of the scrotum by Henry (1946). But as none of the occupations included in the present study, with one exception, shows a very high incidence of cancer of the lung, comparable to that seen in the occupational cancers of the skin and bladder, this source of error is not a very serious one.

(4) *Incorrect diagnosis* is of course a source of error in these, as in all other, medical data. In the case of death certificates received in bulk, perhaps some years after the dates of the deaths recorded, one can do nothing but exclude any certificate of which the wording suggests (1) that the cause of death did not come within the category under examination, or (2) that the diagnosis was uncertain or ill-founded (see next section ; such certificates are made the subject of individual inquiry by the General Register Office). When such exclusions have been made, one must treat the remaining certificates as correct if any use is to be made of the material.

### *Death Certificates Taken for Examination.*

The material utilized in this paper has been restricted as far as possible to cases of primary malignant growth of the lung, and of the larynx. The forms of words in the certificates taken for examination, and in those rejected, may be stated thus :

*Cancer of lung.*

(a) *Retained.*—Cancer, carcinoma, or sarcoma of lung, bronchus, pleura, root of lung, hilum of lung, lung and mediastinum, or lung and pleura. Pulmonary, or bronchial cancer, carcinoma or sarcoma. Cancerous pleurisy.

(b) *Rejected.*—Any new growth of mediastinum, of mediastinal or of bronchial glands. New growths of chest or thorax. Peribronchial new growth. Any new growth qualified by " probable," " doubtful," or " query."

Where another organ is mentioned, in addition to the lung, as the site of new growth, the practice has been to retain those cases where this organ is unlikely to be the primary site (e.g. cancer of lung and brain, or of lung and liver) and to reject the rest (e.g. sarcoma of arm and lung, sarcoma of lung and testicle, cancer of lung and oesophagus).

*Cancer of larynx.*

(a) *Retained.*—The category of cancer of the larynx in the tabulation used in the General Register Office includes malignant growths of which the situation is described by the following terms :

| | |
|---|---|
| Aryteno-epiglottidean folds. | Post-cricoid, retro-cricoid. |
| Arytenoid glands and cartilage. | Pyriform fossa. Sinus pyriformis. |
| Cricoid. | Thyroid cartilage. |
| Epiglottis. | Vallecula epiglottica. |
| Glosso-epiglottic fold. | Vocal cords. |
| Glottis. | Trachea, windpipe. |

All these were retained with the exception of vallecula.

(b) *Rejected.*—Any new growth qualified by "probable," "doubtful," or "query."

Some cases of which the nature is doubtful (e.g. "tumour of cricoid cartilage and thyroid gland").

### *Autopsies.*

The opinion is sometimes expressed that statistics of lung cancer are of no value which comprise cases where no autopsy has been made, and some authorities (see below, p. 289) maintain further that, even when autopsies are performed, many cancers of the lung escape notice. Table II shows the numbers of autopsies recorded in the death certificates for cancer of the lung* discussed here (i.e. after the exclusions stated in the preceding section). Similar data for 1921–32 are given in the earlier paper (Kennaway and Kennaway, 1936). These cases are classified as follows:

No P.M.: (1) No P.M.

(2) Inquest. No P.M.

P.M.: (3) P.M.

(4) Inquest and P.M.

(5) Coroner's P.M. without inquest.

TABLE II.—*Autopsies. Cancer of Lung. (Males,* 1933–38.)

| Year. | I. No P.M. | II. Inquest. No P.M. | III. Sum of I and II. | IV. P.M. | V. Inquest and P.M. | VI. Coroner,s P.M. No inquest. | VII. Sum of IV, V and VI. | Total. |
|---|---|---|---|---|---|---|---|---|
| 1933 | 1,247 | 6 | 1,253 | 439 | 34 | 28 | 501 | 1,754 |
| 1934 | 1,784 | 3 | 1,487 | 494 | 22 | 39 | 555 | 2,042 |
| 1935 | 1,646 | — | 1,646 | 574 | 36 | 34 | 644 | 2,290 |
| 1936 | 1,958 | 4 | 1,962 | 539 | 37 | 34 | 610 | 2,572 |
| 1937 | 2,235 | 5 | 2,240 | 522 | 61 | 40 | 623 | 2,863 |
| 1938 | 2,524 | 5 | 2,529 | 598 | 42 | 53 | 693 | 3,222 |
| Total | 11,094 | 23 | 11,117 | 3166 | 232 | 228 | 3626 | 14,743 |
| 1921–32 | 6,569 | 34 | 6,603 | 2005 | 132 | 68 | 2205 | 8,808 |
| 1921–38 | 17,663 | 57 | 17,720 | 5171 | 364 | 296 | 5831 | 23,551 |

TABLE III.—*Proportion of Autopsies. (Males,* 1921–38.)

| | Total deaths. | Autopsies. | Mean autopsies. Per annum. | Per cent of deaths. |
|---|---|---|---|---|
| Cancer of lung, 1921–32 | 8,803 | 2205 | ·184 | 25·0 |
| ,, ,, 1933–38 | 14,743 | 3626 | 604 | 24·6 |
| ,, of larynx, 1921–32 | 9,472 | 995 | 50 | 6·3 |
| ,, ,, 1933–38 | 5,398 | 448 | 75 | 8·3 |

Table III shows (1) that the number of autopsies has increased much more in the lung series than in the larynx series; and (2) that the average proportion of cases of cancer of the lung examined post mortem is the same in the earlier

* A corresponding table for cancer of the larynx is omitted to save space.

TABLE IV.—*Cancer of Lung.* 1921–1932 *and* 1933–1938.

The occupations are arranged in descending order of the ratio of registered to 100 calculated deaths, 1921–38.

and later periods. Hence the continuing increase in deaths attributed to cancer of the lung is not due to any increase in this proportion.

### COMPARATIVE INCIDENCE UPON VARIOUS OCCUPATIONS.

In the sections below, the periods 1921–32, 1933–38 and 1921–38 are referred to as A, B and C respectively.

The 56 occupations named in Table IV employed during the period B 3,880,001 out of the 13,305,668 males aged 20 and upwards (census of 1931), and they include most of the men engaged in the two largest industries in the country, namely agriculture and coal mining. The tables include 3550 out of the 14,741 cases of cancer of the lung, and 1451 out of the 5397 cases of cancer of the larynx, occurring in period B, in the whole population in question.

Tables IV and V show the data referring to the occupations which have been studied in detail. In Table IV the occupations are arranged in the order of magnitude of the ratio for cancer of the lung, and in Table V in the corresponding order for cancer of the larynx.* The population engaged in each occupation, and the numbers of calculated, and of actual, deaths which are required for the estimation of the ratios are included. The number of deaths in each year of period B in each of these occupations is given in Table XVIII.

### (1) *Agriculture.*

A group of open-air occupations (gardeners, farmers, agricultural labourers, including shepherds, farm bailiffs and foremen), which together include about 954,000 men, has 505 cancers of the lung and 342 cancers of the larynx in B. This group shows very low ratios for lung (from 26 to 58 in A and from 22 to 61 in B) and low ratios for larynx (from 41 to 75 in A and 29 to 79 in B). In this open-air class the highest figure for both lung and larynx is given by gardeners, of whom many live in or near large towns.

### (2) *Coal mining.*

The chief coal-mining occupations (workers above ground, hewers, road-makers, persons conveying material to the shaft, other workers below ground), which employed nearly 770,000 men, show low ratios (lung, 38 to 71 in A and 37 to 72 in B ; larynx, 44 to 58 in A and 42 to 73 in B), which are very like those of the open-air group considered in the preceding paragraph. The air in a coal mine is pumped in from outside, and collieries are not always situated in urban districts (e.g. the Kent coalfield). The close agreement in the ratios given in A and B by the agricultural and coal mining groups is noteworthy.

These two industries (agriculture and coal mining) have also a low incidence of cancer of the bladder (Henry, Kennaway and Kennaway, 1931). The rate of increase of deaths attributed to cancer of the lung in these two groups is shown in Fig. 2.

* The difference between the total numbers of occupations in Table IV (56) and Table V (52) is due to four occupations (patent fuel workers, potters' mill workers, blast furnace engine men, sandblasters) in which no deaths from cancer of the larynx occurred in either period.

(3) *Occupations with exposure to road dust.*

A group of open-air occupations, where there is exposure to the dust of roads, has ratios above 100 for both lung and larynx, with the exception that motor drivers have nearly normal figures for larynx (Table VI). None of these occupations shows a high incidence of cancer of the lung, and the paviours, street masons, concretors and asphalters show a distinct fall in the later period.

(4) *Grooms, horsekeepers, carpenters.*

Grooms and horsekeepers, who are exposed to various forms of dust arising from horses and fodder, have an incidence of cancer of the lung (A 106, B 71) and larynx (A 134, B 100) which is not very far from normal. Carpenters (Table I and Fig. 7) also show low ratios (lung A 73, B 72 ; larynx A 82, B 88). These instances suggest that exposure to dust *per se*, irrespective of its chemical nature, does not conduce to cancer of the lung.

TABLE VI.—*Cancer of Lung and Larynx. Occupations with Exposure to Road Dust.*

| | Ratio. | | | |
|---|---|---|---|---|
| | Lung. | | Larynx. | |
| | 1921–32. | 1933–38. | 1921–32. | 1933–38. |
| Paviours, street masons, concretors and asphalters | 209 | 137 | 154 | 116 |
| Council labourers, road sweepers, dustmen | 173 | 167 | 172 | 267 |
| Drivers of horse-drawn vehicles | 144 | 131 | 184 | 143 |
| Motor drivers (goods and passengers) | 142 | 153 | 100 | 113 |

(5) *Silicosis.*

The occupations in which there is the greatest liability to silicosis are named in Table VII. Of these occupations, those mentioned in Table VIII, and the various subdivisions of coal-mining, were included in the present study. Table VIII shows that in the period 1933–38, the highest incidence of cancer of the lung is in potters' mill workers, slip makers and arkmen (ratio 218), but in this occupation the numbers are small (see Table IV), and hence the sampling error is considerable. In the other occupations in Table VIII the tendency is towards a decrease in the ratio. The stonemasons, and stone miners and quarriers of course include many workers with non-siliceous rocks. The figures for deaths from silicosis in occupations of this class are falling ; in the metal grinders this is due to the adoption of non-siliceous abrasive wheels, which has been advocated by the Senior Medical Inspector of Factories (Factories Report, 1938). In the various groups of coal miners (Table IV) the low ratios show no uniform tendency to rise or fall.* The general indication of these results is that the factors which lead to silicosis are not very active in producing cancer of the lung or larynx. There are, however, in the literature various accounts of cases in which

* C. J. Gooding (*Lancet*, 1946, 251, 891), in a study of pneumoconiosis in South Wales anthracite miners, found no cases of cancer of the lung in 227 necropsies on certified silicotics, and one case associated with silicosis in a further series, making nearly 400 necropsies in all.

cancer of the lung was associated with silicosis. Such individual observations are difficult to assess statistically as the two conditions must sometimes occur together, and the close examination of silicotic lungs in special investigations favours the detection of a high proportion of carcinomas.

TABLE VII.—*Deaths from Silicosis in England and Wales,* 1939 *to* 1945 *inclusive.* (*From the 'Annual Report of the Chief Inspector of Factories for the Year* 1945,' *p.* 79.)

| | | |
|---|---|---|
| 1. | Refractories industries | 68 |
| 2. | Pottery, manufacture of | 310 |
| 3. | Sandstone quarrying and dressing | 173 |
| 4. | Sandstone masons | 363 |
| 5. | Metal grinding | 98 |
| 6. | Sandblasting | 53 |
| 7. | Steel dressing and cleaning of castings | 45 |
| 8. | Stone, pebble, flint and sand crushing | 15 |
| 9. | Scouring powders, manufacture of | 5 |
| 10. | Abrasive wheel manufacture | 4 |
| 11. | Glass cutting and bevelling | 3 |
| 12. | Millstone dressing | 1 |
| 13. | Slate quarrying and dressing | 45 |
| 14. | Granite quarrying and dressing | 13 |
| 15. | Tunnel mining (sewage works, etc.) | 21 |
| 16. | Coal mining | 1731 |

TABLE VIII.—*Cancer of Lung in Occupations with Liability to Silicosis.*

| | Ratios of registered to calculated deaths. | | | Number of deaths. |
|---|---|---|---|---|
| | 1921–32. | 1933–38. | 1921–38. | 1921–38. |
| Potters' mill workers; slip makers and arkmen | 96 | 218 | 174 | 5 |
| Potters; ware-makers, casters and finishers | 152 | 132 | 139·5 | 22 |
| Metal grinders | 229 | 144 | 176 | 39 |
| Stonemasons, cutters and dressers | 149 | 140 | 143 | 86 |
| Stone miners and quarriers | 105 | 49 | 70 | 39 |
| Sand-blasters | 201 | 103 | 136 | 2 |
| Coal-miners (see Table IV) | 58–71 | 37–72 | 43–69 | 774 |

Some of the literature on the relation of silicosis to cancer of the lung is summarized below.

Dible (1934), in the course of 14 autopsies upon persons of both sexes suffering from silicosis, found malignant disease in three cases in stonemasons, as follows:

Case 1. Carcinoma of bile-duct. Spheroidal-cell carcinoma of prostate.
,, 2. Oat-cell carcinoma of lung. Columnar-cell carcinoma of colon.
,, 3. Anaplastic carcinoma of lung.

Anderson and Dible (1938) estimated silica in 48 non-cancerous lungs (A) and 70 lungs showing primary cancer (B). Twelve (C) of B showed abnormal amounts of silica, the mean content of these being nearly 10 times that of A, while the mean content of the remaining 58 (D) of B was the same as that of A. Microscopically, C showed typical silicotic nodules in 7, areas of confluent fibrosis in 3, and fibrosis not more than suggestive of silicosis in 2 of the 12 cases. Fibrosis in A and D " did not in any sense reproduce the picture found in those organs in which silica was present in excess." The authors conclude that " a group of cases of pulmonary carcinoma exists in which the organs contain an excess of silica and show histological evidence of silicotic fibrosis. The conclusion, we think, is that the role of the silicosis is aetiological."

TABLE IX.—*Silicosis and Cancer of Lung* (*Klotz*, 1939).

| | Number of autopsies. | Carcinoma of lung. Number. | Per cent of autopsies. |
|---|---|---|---|
| Cases of silicosis . . | 50 . | 4 | 8·0 |
| Unselected cases . . | 4500 . | 53 | 1·17 |

Klotz (1939) gives some data from Toronto (Table IX) of which the statistical character, and indications, are similar to those of the material of Anderson and Dible.

In the report for 1938 of the Chief Inspector of Factories (p. 81) the Senior Medical Inspector (Dr. J. C. Bridge) gave the following data derived from 1290 autopsies carried out to ascertain whether silicosis was present (Table X). Hence presumably the subjects had shown in life some respiratory symptoms.

TABLE X.—*Silicosis and Cancer of Lung* (*J. C. Bridge*).

| Number of autopsies. | Silicosis. | Cancer of lung. Number of cases. | Per cent of autopsies. |
|---|---|---|---|
| 943 . | Present . | 23 | 2·4 |
| 347 . | Absent . | 17 | 4·9 |
| 1290 | | | |

TABLE XI.—*Cancer of the Lung in Witwatersrand Miners* (*Miners' Phthisis Medical Bureau*).

| European males who had not worked underground (Johannesburg General Hospital). Post mortems. | Cases of primary cancer of lung. | Percentage of all post mortems. | Non-silicotic European miners (Medical Bureau). Post mortems. | Cases of primary cancer of lung. | Percentage of all post mortems. | Silicotic European miners (Medical Bureau). Post mortems. | Cases of primary cancer of lung. | Percentage of all post mortems. |
|---|---|---|---|---|---|---|---|---|
| 1393 | 13 | 0·93 . | 1679<br>1438<br>3117 | 12 | 0·71 . | 1438 | 10 | 0·7 |

The data obtained during 15 years from 3117 post-mortems on European miners on the Witwatersrand (Miners' Phthisis Bureau, 1936) indicate that the incidence of cancer of the lung is the same in silicotic and non-silicotic miners (Table XI).

The account given here of the literature on the relation of silicosis to cancer of the lung does not claim to be complete (see the review by Klotz, 1939).

(6) *Asbestos workers.*

In the years 1939 to 1944 in England and Wales the fatal cases of silicosis and asbestosis numbered 2699 and 75 respectively. The average duration of employment in fatal cases was, in silicosis, 34 years, and in asbestosis 15 years (Factories Report, 1944). In his report for 1938, the Senior Medical Inspector of Factories states that " among 103 fatal cases in which asbestosis or asbestosis with tuberculosis was present, cancer of the lung was associated in 12 cases (11·6 per cent)." Table XII records the cases of cancer of the lung in asbestos workers which have been found in the materials dealt with in this paper.

TABLE XII.—*Cancer of Lung. Asbestos Workers. Males. Death Certificates* 1932–1938.

| | | Age. |
|---|---|---|
| 1935 | Asbestos weaver . . . . . . . | 62 |
| 1935 | ,, cloth weaver . . . . . . | 54 |
| 1938 | ,, weaver . . . . . . . | 46 |
| 1938 | Boiler and pipe asbestos coverer . . . | 58 |
| 1938 | Asbestos packer . . . . . . . | 56 |
| 1936 | Disintegrating room hand in asbestos works . | 65 |
| 1934 | Departmental Manager in asbestos company . | 45 |
| 1936 | Asbestos works manager . . . . | 58 |

These eight cases of cancer of the lung occurred in the years 1934 to 1938 in men of ages from 45 to 65. If one takes the number of deaths from cancer of the lung in these years among the general population of males of approximately the same age groups (45–64) one can make the following comparison :

4,068,382 males (ages 45–64) produced 8191 deaths from cancer of the lung.
= approximately 4 million males (ages 45–64) produced 8000 deaths from cancer of the lung.
= 4000 males produced 8 deaths from cancer of the lung.

The number of male asbestos workers in this age-group appears to be considerably less than 4,000 but as we have been unable to obtain any exact figure for the workers at risk one cannot carry the calculation further.

Lynch and Smith (1935) report from South Carolina a bronchiogenic carcinoma in a man who had been for 21 years an asbestos weaver.

Egbert and Geiger (1936) record a bronchiogenic carcinoma, with the characteristic pulmonary fibrosis and asbestosis bodies, in a man who had been for 18 years an asbestos weaver at New Haven, Connecticut.

Gloyne (1935) records two cases of fairly advanced asbestosis, with squamous carcinoma of the lung, in women workers in England. " One survived nine years after an eight years' exposure to asbestos dust as a spinner ; the other lived for fifteen years after two short periods of six months and thirteen months' exposure in the mattress and opening departments of the factory. . . . The malignant lesions of the lung were, in each case, very small and not recognized during life." Subsequently Gloyne met with asbestosis and an oat-cell carcinoma of the lung in a man who had been for 20 years foreman in the stores department of an asbestos works.

(7) *Alcohol and tobacco.*

There is a considerable difference between occupations associated with the supply of alcohol, and of tobacco ; the former show a very high incidence upon the larynx, and a normal incidence upon the lung (Table XIII). The ratio for the lung is the same for licensed victuallers as for physicians and surgeons (Table XV). The occupations of barman and cellarman show by far the highest figures of any in Table V for cancer of the larynx. The incidence of cancer of the lung upon the two occupations connected with tobacco has decreased distinctly in the later period. The increasing use of cigarettes (Fig. 10, 11) has caused the tobacconist to become more and more a vendor of closed packets which can hardly have any occupational effect. The decline of pipe-smoking makes the mixing by the tobacconist by hand of a blend to suit a customer's taste to be less frequent.

TABLE XIII.—*Cancer of Lung and Larynx. Occupations Associated with the Supply of Alcohol and Tobacco.*

| | Ratio. | | | |
|---|---|---|---|---|
| | Lung. | | Larynx. | |
| | 1921–32. | 1933–38. | 1921–32. | 1933–38. |
| Barmen | 104 | 122 | 469 | 275 |
| Cellarmen | 92 | 87 | 317 | 502 |
| Licensed victuallers | 128 | 124 | 185 | 199 |
| Tobacco manufacturers | 196 | 110 | 65 | 129 |
| Tobacconists and their assistants | 175 | 82·5 | 142 | 171 |

(8) *Coal-gas and tar.*

The occupations associated with coal-gas and tar are important on account of the known liability of the workmen to cancer of the skin, and of the bladder (Henry, Kennaway and Kennaway, 1931), but they offer unsatisfactory statistical material because some of the most important occupations employ very few men, and hence produce but few cases of cancer, so that the sampling error is high even over an 18-year period. This difficulty can only be met by omitting some of the smaller classes. Thus, in Table XIV there are ten occupations in which there is especial exposure to coal-gas and tar. If from these ten one excludes the four which have produced less than ten deaths from cancer of the lung, one obtains, by this quite arbitrary procedure, the results shown in Table XIV.

Thus all the six occupations retained, which employ about 82,000 men (1931 census) and yield 267 cases of cancer of the lung, have an increased (up to 284) ratio for this disease, the lowest figure being that for chimney sweeps (119).

TABLE XIV.—*Cancer of Lung. Occupations Associated with Coal Gas and Tar.*

| | 1921–38, deaths. | Ratio. 1921–32. | 1933–38. | 1921–38. |
|---|---|---|---|---|
| Gas stokers and coke-oven chargers | 85 | 342 | 251 | 284 |
| Gas producermen | 12 | 162 | 232 | 202 |
| Gas-works foremen and inspectors | 25 | 197 | 161 | 174 |
| Gas fitters | 41 | 182 | 158 | 167 |
| Gas-works' labourers | 96 | 162 | 109 | 129 |
| Chimney sweeps | 21 | 170 | 90 | 119 |
| | 267 | | | |
| Excluded : | | | | |
| Labourers, patent fuel works | 3 | 433 | 1529 | 571 |
| Gas-works engine and crane drivers | 6 | 60 | 186 | 138 |
| Gas-works managers | 4 | 244 | 58 | 136 |
| Tar distillery workers and coke-oven workers | 8 | 29 | 70 | 52 |
| | 21 | | | |

In five of the six cases, and especially in sweeps, the ratio has fallen in the later period. The ratios for cancer of the larynx are irregular throughout this class.

(9) *Professional occupations.*

A group of professional workers (stockbrokers and stockjobbers ; clergymen ; Roman Catholic priests and monks ; ministers of other religious bodies ; judges, stipendiary magistrates and barristers ; solicitors ; physicians, surgeons and registered medical practitioners ; dental practitioners) numbering over 108,000 men and showing 176 deaths from cancer of the lung and 39 from cancer of the larynx, give altogether ratios rather below the average (lung A 94, B 97 ; larynx A 84, B 50 ; Tables IV, V and XV). These ratios for cancer of the lung, being near 100, are in accordance with Stevenson's (1923) discovery of the absence of social gradient in this form of cancer. When the individual occupations are examined considerable variations are found. In the earlier paper attention was drawn to the high ratio for cancer of the lung in judges, stipendiary magistrates and barristers (173) and in stockbrokers and stockjobbers (187) ; in the later period (1933–38) the former fell to 112 and the latter rose to 253. The stockbrokers and jobbers also have the highest ratio among the professional workers for cancer of the larynx. The figures for Roman Catholic priests and monks are too small to be significant. The ratio for ministers of other religious bodies has risen (Table XX). In regard to the view that the rapid increase in recorded deaths from cancer of the lung is due to the detection of more cases by improved diagnosis, the very moderate and consistent ratio (A 129, B 125) for cancer of the lung in medical men is noteworthy, for this is an occupation where the availability of the existing methods for the detection of cancer is presumably at a maximum. Dental practitioners give very similar figures (A 103, B 124).

The ratios for cancer of the larynx in these occupations are below, and in some cases considerably below, 100, except in stockbrokers and stockjobbers (158).

TABLE XV.—*Cancer of Lung. Professional Occupations.*

| Occupation. | 1921–32. Population. | 1921–32. Total regd. deaths. | 1921–32. Ratio of regd. to 100 calculated deaths. | 1933–38. Population. | 1933–38. Total. regd deaths. | 1933–38. Ratio of regd. to 100 calculated deaths. | 1921–38. Ratio of regd. to 100 calculated deaths. | 1933–38. Cancer of larynx. Deaths. | 1933–38. Cancer of larynx. Ratio. |
|---|---|---|---|---|---|---|---|---|---|
| Stockbrokers and stockjobbers | 6,893 | 14 | 187 | 7,122 | 32 | 253 | 229 | 8 | 158 |
| Roman Catholic Priests and Monks | 3,604 | 1 | 31 | 4,050 | — | — | 11 | 5 | 24 |
| Clergymen (Anglican Church) | 23,422 | 15 | 53 | 22,754 | 22 | 49 | 50 | 1 | 45·5 |
| Ministers of other Religious Bodies | 11,769 | 5 | 38 | 11,960 | 15 | 69 | 57 | 6 | 64 |
| Judges, Stipendiary Magistrates and Barristers | 3,658 | 7 | 173 | 3,632 | 7 | 112 | 136 | 1 | 34·5 |
| Solicitors | 17,306 | 19 | 100 | 17,853 | 26 | 84 | 90 | 9 | 65·5 |
| Physicians, Surgeons and Registered Medical Practitioners | 27,951 | 35 | 129 | 29,908 | 56 | 125 | 127 | 7 | 38 |
| Dental Practitioners | 10,494 | 8 | 103 | 11,633 | 18 | 124·5 | 117 | 2 | 44 |
| | | | | | | | | 39 | |

(10) *Cotton workers.*

Cotton strippers, grinders and card-room jobbers, among whom severe bronchitic and asthmatic affections occur, show a very low ratio (30) for cancer of the lung in A, which rose to the normal level (104) in B (see p. 286).

Cotton spinners and piecers have a low but increasing ratio for lung (A 32, B 75) and medium ratio for larynx (A 148, B 91). The total population of 43,991 (1931 census) includes ring spinners as well as mule spinners, but the latter occupation certainly makes up a very large proportion of the total, for the number of mule spinners aged 20 and over was estimated in 1926 as 41,000 by the Departmental Committee appointed to consider evidence as to the Occurrence of Epitheliomatous Ulceration among Mule Spinners. In view of the exposure to oils which may be carcinogenic the low incidence of cancer of the lung is remarkable, but the very high incidence of cancer of the skin, and the increased liability to cancer of the bladder (Henry, Kennaway and Kennaway, 1931) in this occupation, must be taken into account. The question of the penetration into the lung of oil droplets in the air of the mule room will be dealt with in another publication.

### RATE OF INCREASE OF CANCER OF THE LUNG AND LARYNX IN MEN AND WOMEN.

*Cancer of lung.*

Cancer of the lung has increased from 361 to 5982 cases per annum in men (Fig. 1) between 1921 and 1945, and from 186 to 1480 cases in women. These figures are in the ratio of 1 : 16·6 (men) and 1 : 8 (women). The increase became more rapid in 1926 and again in 1930 in men (Fig. 1) and about 1929 in women. The annual increase, in men, between 1932 and 1937 was at the rate of from 200 to 300 cases per annum ; since then it has been very irregular

(Table XVI). The ratio between deaths from cancer of the lung in men, and in women, is now (i.e. in 1945, the latest year for which figures are available) 1 : 4·0, which is approaching the latest available (1939) ratio for cancer of the mouth (Table XVII). Cancer of the lung differs from the other forms of cancer in Table XVII in that the proportion of males is increasing. During the whole

Fig. 3.—Deaths from cancer of lung. Males and Females. 1935–1945.

period under consideration here (1921–1945) the ratio of female to male deaths from cancer of the lung has changed from 1 : 1·9 to 1 : 4·0. A similar change has occurred in the United States ; the ratio of the standardized death rates for cancer of the lung and pleura in white men and women was 3 : 1 in 1938, and 20 years earlier was less than 1·5 : 1 (Statistical Bulletin, 1939).

Fig. 3 shows that the rate of increase in recent years of deaths attributed to cancer of the lung is much greater in men than in women, for the curve of deaths of males rises much more steeply than does that of deaths of females. But this apparent great difference between the sexes is due in large part to the fact that, in a graph of this type, a given increase in a larger quantity is much more conspicuous than is the same percentage increase in a smaller quantity. If one reckons the annual numbers of deaths as a percentage of those in any given initial year (Table XVI), the difference between the two sexes is not nearly so conspicuous (Fig. 4).

Fig. 4.—Cancer of lung. Males and Females. 1935–1945. Deaths per annum as percentage of deaths in 1935.

Table XVI.—*Totals of Death Certificates (England and Wales) and Percentage Increase in Cancer of the Lung.*

| | Cancer of lung. | | Cancer of larynx. | |
|---|---|---|---|---|
| | Men. | Women. | Men. | Women. |
| 1935 | 2337 = 100 | 858 = 100 | 903 | 224 |
| 1936 | 2606 = 112 | 917 = 107 | 916 | 275 |
| 1937 | 2914 = 126 | 927 = 108 | 918 | 232 |
| 1938 | 3273 = 140 | 1049 = 122 | 887 | 259 |
| 1939 | 3391 = 145 | 1149 = 134 | 929 | 280 |
| 1940 | 3808 = 164 | 1180 = 137 | 903 | 267 |
| 1941 | 4069 = 174 | 1156 = 135 | 929 | 269 |
| 1942 | 4579 = 196 | 1267 = 148 | 857 | 255 |
| 1943 | 5081 = 217 | 1296 = 151 | 972 | 281 |
| 1944 | 5491 = 235 | 1360 = 158 | 852 | 280 |
| 1945 | 5982 = 256 | 1480 = 172 | 841 | 279 |

TABLE XVII.—*Cancer of Lung, Oesophagus and Mouth Region in Men and Women (England and Wales).*

| | Site of cancer. | Number of deaths. Women. | Men. | Ratio. |
|---|---|---|---|---|
| 1921 | Lung | 186 | 361 | 1 : 1·9 |
| 1932 | ,, | 565 | 1553 | 1 : 2·7 |
| ,, | Oesophagus | 661 | 1774 | 1 : 2·7 |
| ,, | Mouth region (lip, tongue, mouth, tonsil, jaw, pharynx) | 536 | 3040 | 1 : 5·7 |
| 1937 | Lung | 927 | 2914 | 1 : 3·1 |
| 1939 | ,, | 1029 | 3544 | 1 : 3·4 |
| ,, | Oesophagus | 729 | 1590 | 1 : 2·2 |
| ,, | Mouth region | 585 | 2538 | 1 : 4·3 |
| 1945 | Lung | 1480 | 5982 | 1 : 4·0 |

*Cancer of larynx.*

Cancer of the larynx has increased between 1921 and 1945 from 641 to 841 cases per annum in men, with a maximum of 972 in 1943, and from 138 to 279 cases in women (Table XVI). These figures are in the ratio of 1 : 1·3 in men and 1 : 2·0 in women. But the figures for men have been fluctuating in the region between 850 and 970 since 1930, and in women there has been no uniform increase since 1928. Hence if the adoption of smoking by women has had any effect upon their liability to the disease, this factor has reached equilibrium.

### COMPARISON OF THE RATIOS IN THE EARLIER AND LATER PERIODS.

The calculation of these ratios (Tables IV and V) for the two periods 1921–32 (A) and 1933–38 (B) provides material for a comparison. If the data from which the ratios are derived are reliable, this comparison should show whether the incidence of cancer of the lung or larynx upon any given occupation has undergone changes differing significantly from any which have occurred in the general population, and the general degree of agreement between the ratios for the two periods is some indication of the value of conclusions based on death certificates. In Fig. 5 the comparison is made for cancer of the lung by calculating the ratio between the earlier and later ratios (B/A × 100) and setting down the figures in order of magnitude. Occupations with not more than one death in either period are omitted here, and are considered elsewhere (p. 286). The results shown in the graph may be summarized thus :

| | |
|---|---|
| Ratios below 66 | 11 |
| ,, 66 to 133 | 35 |
| ,, above 134 | 3 |
| | 49 |

Thus 35/49 of the ratios of one period, or 71·4 per cent, are within ± 33 per cent of those of the other, which is perhaps as close an agreement as one could look

FIG. 5.—Cancer of lung. Males in 49 occupations.
A. Ratio of registered to 100 calculated deaths, 1921–1932.
B. Ratio of registered to 100 calculated deaths, 1933–1938.
Dots represent B/A × 100.
Occupations with not more than one death in either period are omitted.

for in such material. When the individual points on the graph are examined, the larger occupations are found to lie chiefly in the middle part of the series, as one would expect from the lower sampling error.

A similar treatment of the figures for cancer of the larynx (Fig. 6) gives the following result :

| | |
|---|---|
| Ratios below 66 . . . | 7 |
| ,, 66 to 133 . . . | 28 |
| ,, above 144 . . . | 14 |
| | 49 |

In this case 28/49 or 57 per cent of the earlier ratios are within ± 33 per cent of the later ones. There are thus more occupations in which the ratio has increased than in the case of cancer of the lung.

OCCUPATIONAL INCIDENCE OF THE INCREASE OF CANCER OF THE LUNG.

If there is a real increase in cancer of the lung, and if this is due to some external factor, one might find differences in the rate of increase in classes of persons exposed to different environments. The annual numbers of deaths from cancer of the lung, and of the larynx, in each one of the occupations studied in detail in this paper can be seen in Table XVIII. The increase in the total of recorded cases has been so great (Fig. 1) that, if this increase were due to a contribution from any of the smaller occupations, the incidence upon these would have to be very high indeed.

TABLE XVIII.—*Number of Deaths from Cancer of the Lung and Larynx in 56 Occupations in each Year* 1933–1938.

The occupations are placed in the same order as in Table IV.

| | | 1933. | 1934. | 1935. | 1936. | 1937. | 1938. | Total. |
|---|---|---|---|---|---|---|---|---|
| Labourers, patent fuel works | Lung | — | — | — | — | — | 1 | 1 |
| | Larynx | — | — | — | — | — | — | — |
| Gas stokers and coke-oven chargers | Lung | 9 | 5 | 4 | 8 | 11 | 11 | 48 |
| | Larynx | 2 | 3 | 3 | 4 | 2 | 3 | 17 |
| Gas producer men | Lung | 1 | — | 4 | — | — | 3 | 8 |
| | Larynx | — | — | — | — | — | — | — |
| Metal grinders | Lung | 1 | 3 | 4 | 1 | 5 | 6 | 20 |
| | Larynx | 3 | 2 | — | 1 | 2 | 1 | 9 |
| Gas-works foremen and inspectors | Lung | 2 | 2 | 3 | 5 | — | 3 | 15 |
| | Larynx | — | — | — | 1 | 1 | — | 2 |
| Potters' mill workers; slip makers and arkmen. | Lung | — | 1 | 1 | 1 | 1 | — | 4 |
| | Larynx | — | — | — | — | — | — | — |
| Mainly council labourers, road sweepers and dustmen | Lung | 18 | 10 | 16 | 18 | 16 | 25 | 103 |
| | Larynx | 6 | 7 | 9 | 16 | 12 | 13 | 63 |
| Gas fitters | Lung | — | 4 | 4 | 8 | 3 | 6 | 25 |
| | Larynx | 1 | — | 1 | 2 | — | 2 | 6 |
| Paviours, street masons, concretors and asphalters | Lung | 4 | 3 | 2 | 1 | 2 | 2 | 14 |
| | Larynx | — | 1 | 1 | 2 | — | — | 4 |
| Motor drivers, goods and passengers | Lung | 30 | 29 | 42 | 43 | 72 | 85 | 301 |
| | Larynx | 7 | 6 | 8 | 8 | 8 | 11 | 48 |
| Blast furnace engine men | Lung | — | — | 1 | — | — | — | 1 |
| | Larynx | — | — | — | — | — | — | — |
| Cabinet makers | Lung | 10 | — | 9 | 14 | 14 | 13 | 60 |
| | Larynx | 5 | 2 | 5 | 6 | — | 2 | 20 |
| Tobacco manufacturers | Lung | 3 | 2 | — | 1 | — | 2 | 8 |
| | Larynx | — | — | 3 | 1 | — | — | 4 |
| Stone masons, cutters and dressers | Lung | 11 | 15 | 13 | 16 | 17 | 13 | 85 |
| | Larynx | 1 | 1 | 4 | 3 | 2 | 1 | 12 |
| Tanners, leather dressers, curriers, skilled workers | Lung | 4 | 5 | 3 | 5 | 4 | 9 | 30 |
| | Larynx | — | 1 | 2 | — | 2 | — | 5 |
| Potters; ware-makers, casters and finishers | Lung | 1 | 2 | 1 | 1 | 5 | 3 | 13 |
| | Larynx | — | 1 | — | — | 1 | 2 | 4 |
| Gas-works engine and crane drivers | Lung | — | 1 | 2 | 1 | — | 1 | 5 |
| | Larynx | — | 1 | — | — | 1 | — | 2 |
| Sandblasters | Lung | — | — | 1 | — | — | — | 1 |
| | Larynx | — | — | — | — | — | — | — |
| Gas-works managers | Lung | — | — | — | — | — | 1 | 1 |
| | Larynx | — | 1 | — | — | — | — | 1 |
| Drivers of horse-drawn vehicles | Lung | 25 | 40 | 35 | 51 | 45 | 44 | 240 |
| | Larynx | 18 | 17 | 23 | 17 | 18 | 7 | 100 |
| Gas-works labourers | Lung | 4 | 8 | 8 | 9 | 9 | 12 | 50 |
| | Larynx | 1 | 1 | 3 | 5 | 1 | — | 11 |
| French polishers | Lung | 5 | 2 | 4 | 4 | 2 | 4 | 21 |
| | Larynx | 2 | 2 | 4 | — | — | 1 | 9 |
| Painters | Lung | 29 | 47 | 43 | 51 | 76 | 77 | 323 |
| | Larynx | 27 | 24 | 20 | 11 | 18 | 20 | 120 |
| Licensed victuallers | Lung | 18 | 33 | 21 | 29 | 38 | 33 | 172 |
| | Larynx | 17 | 21 | 14 | 22 | 14 | 14 | 102 |
| Printers | Lung | 14 | 13 | 23 | 14 | 26 | 26 | 116 |
| | Larynx | 6 | 4 | 6 | 6 | 7 | 4 | 33 |
| Chimney sweeps | Lung | — | — | 3 | 1 | 2 | 4 | 10 |
| | Larynx | 1 | — | 1 | 1 | 2 | 1 | 6 |
| Plumbers | Lung | 5 | 10 | 8 | 12 | 14 | 17 | 66 |
| | Larynx | 3 | 4 | 2 | 5 | 4 | 4 | 22 |
| Barmen | Lung | 2 | 1 | 6 | 4 | 2 | 5 | 20 |
| | Larynx | 3 | 3 | 2 | 2 | 1 | 2 | 13 |

TABLE XVIII (*contd.*).

| | | 1933. | 1934. | 1935. | 1936. | 1937. | 1938. | Total. |
|---|---|---|---|---|---|---|---|---|
| Tobacconists and their assistants | Lung | 1 | 4 | 1 | 5 | 3 | 3 | 17 |
| | Larynx | 3 | 3 | 3 | — | 3 | 2 | 14 |
| Hairdressers | Lung | 5 | 8 | 4 | 11 | 6 | 13 | 47 |
| | Larynx | 3 | 2 | 3 | — | — | 2 | 10 |
| Bakers and pastry cooks | Lung | 10 | 9 | 12 | 15 | 12 | 18 | 76 |
| | Larynx | 3 | 7 | 2 | 4 | 5 | 3 | 24 |
| Professional men | Lung | 25 | 29 | 27 | 34 | 26 | 35 | 176 |
| | Larynx | 8 | 3 | 4 | 9 | 10 | 5 | 39 |
| Cellarmen | Lung | 2 | — | — | 1 | 1 | 2 | 6 |
| | Larynx | 2 | 2 | 3 | 1 | 2 | 2 | 12 |
| Pottery, etc., kiln and ovenmen, kiln setters and placers | Lung | 1 | 1 | 2 | 2 | 3 | 3 | 12 |
| | Larynx | — | 5 | — | 2 | 1 | 2 | 10 |
| Grooms and horsekeepers | Lung | 3 | 8 | 5 | 5 | 2 | 7 | 30 |
| | Larynx | 4 | 2 | 4 | — | 5 | 2 | 17 |
| Brick kiln and ovenmen | Lung | 3 | — | 2 | 1 | — | 3 | 9 |
| | Larynx | — | 2 | — | — | 1 | — | 3 |
| Stationary engine and crane drivers | Lung | 9 | 9 | 15 | 11 | 14 | 17 | 75 |
| | Larynx | 7 | 4 | 6 | 2 | 2 | 3 | 24 |
| Cotton strippers, grinders and card room jobbers | Lung | — | — | — | 2 | 1 | 3 | 6 |
| | Larynx | — | — | — | — | 1 | 1 | 2 |
| Locomotive engine drivers, firemen and cleaners | Lung | 11 | 6 | 10 | 13 | 13 | 13 | 66 |
| | Larynx | 3 | 5 | 1 | 2 | 5 | 2 | 18 |
| Carpenters | Lung | 25 | 34 | 35 | 31 | 35 | 40 | 200 |
| | Larynx | 21 | 14 | 17 | 18 | 15 | 12 | 97 |
| Stone miners and quarriers | Lung | 2 | 2 | 1 | 5 | 3 | 4 | 17 |
| | Larynx | 6 | 2 | 2 | 1 | 1 | — | 12 |
| Coal miners—hewers and getters | Lung | 51 | 49 | 51 | 40 | 62 | 66 | 319 |
| | Larynx | 20 | 14 | 24 | 14 | 20 | 15 | 107 |
| Lithographic and process engravers | Lung | 2 | 1 | — | — | — | 1 | 4 |
| | Larynx | — | 2 | — | 1 | 2 | — | 5 |
| Blast furnacemen and labourers | Lung | — | — | 1 | 1 | 1 | 1 | 4 |
| | Larynx | 1 | — | — | 1 | 1 | 1 | 4 |
| Gardeners | Lung | 21 | 31 | 38 | 40 | 41 | 42 | 213 |
| | Larynx | 16 | 23 | 11 | 25 | 20 | 28 | 123 |
| Cotton spinners and piecers (mule, ring, cap or flyer) | Lung | 4 | 4 | 3 | 6 | 7 | 3 | 27 |
| | Larynx | 1 | 2 | 2 | 3 | — | 3 | 11 |
| Coal miners—workers above ground | Lung | 6 | 11 | 5 | 11 | 7 | 9 | 49 |
| | Larynx | 7 | 2 | 3 | 5 | 3 | 6 | 26 |
| Tar distillery workers and coke-oven workers | Lung | 1 | 2 | 2 | — | — | 1 | 6 |
| | Larynx | — | — | — | — | — | — | — |
| Brick and plain tile makers | Lung | — | — | 2 | 1 | 2 | — | 5 |
| | Larynx | — | — | — | — | — | — | — |
| Coal miners—other workers below ground | Lung | 6 | 7 | 10 | 10 | 7 | 12 | 52 |
| | Larynx | 2 | 1 | 4 | 5 | 2 | 4 | 18 |
| Coal miners—conveying material to the shaft | Lung | 3 | 2 | 5 | 3 | 4 | 11 | 28 |
| | Larynx | 2 | — | — | — | 1 | 4 | 7 |
| Coal miners—making roads | Lung | 6 | 11 | 2 | 7 | 4 | 7 | 37 |
| | Larynx | 2 | 4 | 4 | 8 | 3 | 3 | 24 |
| Cotton weavers | Lung | 3 | 5 | 3 | 3 | 3 | — | 17 |
| | Larynx | 3 | — | 1 | 2 | 2 | 4 | 12 |
| Farmers | Lung | 17 | 24 | 21 | 29 | 27 | 30 | 148 |
| | Larynx | 20 | 12 | 8 | 14 | 16 | 11 | 81 |
| Agricultural labourers, including shepherds | Lung | 17 | 21 | 17 | 31 | 23 | 28 | 137 |
| | Larynx | 21 | 19 | 28 | 23 | 22 | 22 | 135 |
| Farm bailiffs and foremen | Lung | 2 | — | 1 | 1 | 1 | 1 | 6 |
| | Larynx | 1 | — | 2 | — | — | — | 3 |
| | | | | | Lung | | | 3550 |
| | | | | | Larynx | | | 1451 |

FIG. 6.—Cancer of larynx. Males in 49 occupations.
A. Ratio of registered to 100 calculated deaths, 1921–1932.
B. Ratio of registered to 100 calculated deaths, 1933–1938.
Dots represent B/A × 100.
Occupations with not more than one death in either period are omitted.

TABLE XIX.—*Increase of Deaths Attributed to Cancer of Lung in Certain Occupations* (1927–1938).

| | | Deaths from cancer of lung. | | |
|---|---|---|---|---|
| | | 1927–32. | 1933–38. | Difference. |
| See Table VI | Paviours, street masons, concretors, asphalters | 7 | 14 | 7 |
| | Council labourers, road sweepers, dustmen | 47 | 103 | 56 |
| | Drivers of horse-drawn vehicles | 111 | 240 | 129 |
| | Motor drivers | 102 | 301 | 199 |
| | | 267 | 658 | 391 |
| General population, males | | 6296 | 15,045 | 8749 |

For example, if one takes the numbers of deaths from cancer of the lung in those occupations where there is especial exposure to road dust (Table VI) and divides them between two successive periods of 6 years (1927–32 and 1933–38) and compares these figures with those given by the whole male population, it becomes quite clear that even these occupations account for only a small fraction (391 cases or 4·5 per cent) of the whole increase of 8749 cases (Table XIX).

The following example shows the effect of the increase in deaths attributed to cancer of the lung upon the data for a single occupation.

210,431 carpenters produced 123 deaths from cancer of the lung in 12 years (1921–33) or 10·2 deaths per annum, and in the next 6 years (1933–38) 243,928 carpenters produced 200 such deaths, or 33·3 deaths per annum (Table IV). The number of carpenters producing 1 death in 1 year was therefore 210,431 ÷ 10·2 = 20,640 in the first period and 243,928 ÷ 33·3 = 7324 in the second. The ratio between the two figures, namely 3·3 : 1, is reasonably near the inverse of the ratio (1 : 2·8) between the mean annual deaths (755 and 2507) in these two periods from cancer of the lung in the whole male population. The result indicates that the increase of cancer of the lung has fallen upon carpenters, who undergo considerable exposure to some kinds of dust, in much the same measure as it has upon men in general (Fig. 7).

FIG. 7.—Deaths from cancer of lung. Carpenters, 1921–1938.

Occupational exposure to three agents which are perhaps liable to induce cancer of the lung (asbestos, arsenic, nickel [Factories Report, 1938, p. 72]) occurs in employments which comprise very few workers, and hence have no appreciable effect upon the total. Of these three substances, arsenic seems to be the one to which the general population is most likely to be exposed. No attempt is made here to deal with all the data on the carcinogenic action of arsenic, as this subject is under investigation by a Committee of the Industrial Health Board, but the following quotations from recent Annual Reports of the Senior Medical Inspector of Factories show the possible importance of arsenic in relation to cancer of the lung. " In the case of arsenical poisoning due to sodium arsenite, which was fatal, the post-mortem examination revealed that in addition to pigmentation of the trunk and limbs, warty growths all over the body, and perforation of the nasal septum, there was a primary cancer of the right lung with metastatic growths in neighbouring glands and in the liver " (Factory Report, 1939, p. 22). " The other (case) occurred in a filling machine operator aged 57, for 43 years in a factory manufacturing sheep dip containing sodium arsenite, the cause of death being due to carcinoma of the right lung."

" Three similar cases of pulmonary carcinoma occurring in arsenical sheep-dip workers have been notified since 1939 " (Factory Report, 1943, p. 45).

Neubauer (1947) collected the literature relating to 142 cases of medicinal arsenical epithelioma ; in one of these cancer of the lung occurred (Montgomery, 1935 ; Montgomery and Waisman, 1941). The patient had taken a medicine for eczema for six years in childhood, which was assumed to contain arsenic on account of the appearance from the age of 42 onwards of numerous epitheliomas of the skin of the arsenical type. Later an intraurethral epithelioma developed, and finally an epithelioma of the bronchus with " malignant dyskeratosis characteristic of the arsenical type of epithelioma," and extensive metastases.

Semon (1945) records a case of bronchial carcinoma in a man who, during a period of about 20 years, had taken large doses—up to 1 drachm 3 times daily—of Fowler's solution (equivalent to 1 per cent arsenic trioxide) for recurrent attacks of severe dermatitis herpetiformis. His skin was unaffected. There is, of course, nothing to show that the bronchial carcinoma was due to the arsenic, but unless attention is drawn to such a case others may not be noticed.

TABLE XX.—*Cancer of the Lung. Occupations Showing a Considerable Rise in the Ratio in the Later Period.*

| | 1921–32. A. | | | 1933–38. B. | | | |
|---|---|---|---|---|---|---|---|
| | Population. | Number of deaths. | Ratio. | Population. | Number of deaths. | Ratio. | Ratio B/A × 100. |
| Potter's mill workers, slip-makers and arkmen | 1,486 | 1 | 96 | 1,766 | 4 | 218 | 227 |
| Gasworks engine and crane drivers | 1,854 | 1 | 60 | 1,800 | 5 | 186 | 310 |
| Cotton spinners and piecers | 38,817 | 7 | 32 | 43,991 | 27 | 75 | 234 |
| Cotton strippers and grinders and card room jobbers | 5,242 | 1 | 30 | 5,342 | 6 | 104 | 347 |
| Tar distillery workers and coke-oven workers | 9,697 | 2 | 29 | 7,824 | 6 | 70 | 242 |
| Brick kiln and ovenmen | 6,225 | 1 | 23 | 7,112 | 9 | 121 | 528 |
| Ministers of other religious bodies | 11,769 | 5 | 38 | 11,960 | 15 | 69 | 182 |

(1) *Cancer of the lung.*

In 7 occupations the ratios for B are higher than those for A by more than 50 per cent (Table XX and Fig. 5). In all of these the ratios in A are lower, and in most cases much lower, than 100, and hence the considerable rise raises the ratio in B above 150 in two cases only (potter's mill workers, slip makers and arkmen 218; gas-works engine and crane drivers 186). In 4 of the 7 occupations there is only one death in A, and hence the comparison of the two periods is liable to considerable sampling error. The fact that the rise starts from a low level does not, of course, prove that it is unimportant, and these occupations must be watched in the subsequent years to see if the rise continues.

Cases of cancer of the lung have occurred among stokers of oil-fired boilers, but no figure for the population of these workers is available.

(2) *Cancer of the larynx.*

In 9 occupations the ratios for B are higher than those for A by more than 50 per cent (Table XXI and Fig. 6). In 6 of these there are not more than 3 deaths in the first period, so that the sampling error is high, and the ratio in the first period is low.

TABLE XXI.—*Cancer of the Larynx. Occupations Showing a Considerable Rise in the Ratio in the Later Period.*

| | 1921–32. A. | | | 1933–38. B. | | | |
|---|---|---|---|---|---|---|---|
| | Population. | Number of deaths. | Ratio. | Population. | Number of deaths. | Ratio. | Ratio B/A × 100. |
| Cellarmen | 6,333 | 14 | 317 | 7,050 | 12 | 501 | 158 |
| Metal grinders | 15,220 | 10 | 141 | 17,009 | 9 | 234 | 166 |
| Brick-kiln and oven men | 6,225 | 3 | 71 | 7,112 | 3 | 123 | 173 |
| Pottery, etc., kiln and oven men | 12,188 | 9 | 116 | 14,442 | 10 | 220·5 | 190 |
| Cotton strippers and grinders and card room jobbers | 5,242 | 2 | 60 | 5,342 | 2 | 118 | 197 |
| Tobacco manufacturers | 7,698 | 3 | 65 | 8,192 | 4 | 129 | 198·5 |
| Gas works managers | 1,303 | 1 | 76 | 1,049 | 1 | 157 | 207 |
| Lithographic and process engravers | 5,704 | 3 | 84 | 7,289 | 5 | 214 | 255 |
| Blast furnacemen and labourers | 9,391 | 3 | 40 | 6,135 | 4 | 138 | 346 |

### ERRORS IN THE DIAGNOSIS OF CANCER OF THE LUNG.

Some hold that cancer of the lung is not increasing, and that the ascent of the curve (Fig. 1) is due to improved diagnosis.

This statement is easy to make and difficult, perhaps impossible, to disprove, and is apt to serve as a substitute for any investigation of the question. If one accepts it, at least one conclusion follows which is of some interest. Let us suppose that the steeply rising line (Fig. 1) represents the detection of an increasing fraction of a pool of cases awaiting discovery. When the means of diagnosis, and their availability, can be improved no further, the curve must flatten. We cannot say when this will happen, so for the present we must take the latest, and highest, figure (5982 deaths of males in 1945) as representing the total. If the actual number has altered little during recent years a similar number of deaths took place in, say, 1921, but only 361 of these deaths (Fig. 1) were assigned to the right cause. Hence in that year in at least 5982 — 361 = 5621 fatal cases of cancer of the lung the cause of death entered upon the death certificate was wrong. This is of course possible, but it would be interesting to know what were the 5621 erroneous descriptions of *fatal* disease.

Some conditions which might give rise to false diagnoses in cases of cancer of the lung are* :

(1) *Mediastinal tumour.*—The increase of cases of cancer of the lung cannot depend wholly, or even largely, upon a diversion to that category of cases formerly set down as mediastinal tumour because the number of these has never been sufficient.

Thus the death certificates for males show no fall in deaths attributed to cancer of the mediastinum during the years following 1931 (when this form of cancer is first mentioned in the Registrar-General's Statistical Reviews), which would account for the increase of 7024 in cases of cancer of the lung which took place in the years 1931–37 (Table XXII).

* I am indebted to Professor D. W. Smithers, Professor R. A. Willis and Dr. Eric Scowen for information on this subject.

TABLE XXII.—*Cancer of Lung and of Mediastinum.*

| | Deaths of males. England and Wales. Cancer of lung. | Deaths of males. England and Wales. Cancer of mediastinum.* |
|---|---|---|
| 1931 | 1,055 | 135 |
| 1932 | 1,553 | 262 |
| 1933 | 1,820 | 262 |
| 1934 | 2,095 | 244 |
| 1935 | 2,345 | 248 |
| 1936 | 2,611 | 222 |
| 1937 | 2,930 | 308 |
| Total | 14,409 | 1681 |
| 7 × 1055 | 7,385 | |
| Increase | 7,024 | |

* These figures are taken from the Registrar-General's Statistical Reviews for the years in question.

(2) *Pulmonary infections.*—A long list could be compiled of infective processes and their results which might have to be considered in the diagnosis of cancer of the lung in the living subject, but those most likely to be named in error on death certificates are tuberculosis, lung abscess, pneumonia, and bronchiectasis. In theory, the numbers of deaths attributed to these conditions should be falling if increasing numbers of cancers of the lung are being detected among such cases. But actually the numbers of deaths attributed to respiratory tuberculosis, and to pneumonia, are so large, ranging from 2 to 10 times those assigned to cancer of the lung, that the proportional fluctuations prevent any diminution which could be due to this cause from being identified (Fig. 8). In this figure the two curves representing pulmonary infections show no uniform relationship to the third representing cancer of the lung. The numbers of deaths from abscess of the lung (Table XXIII) are too small (mean 100 per annum) to be of importance, and moreover they show no continuous fall during the period. Bronchiectasis is not separated from bronchitis in the Registrar-General's Statistical Reviews.

TABLE XXIII.—*Deaths of Males from Abscess of Lung. England and Wales.*

| | | | |
|---|---|---|---|
| 1932 | 92 | 1937 | 96 |
| 1933 | 82 | 1938 | 132 |
| 1934 | 91 | 1939 | 119 |
| 1935 | 111 | 1940 | 84 |
| 1936 | 102 | 1941 | 92 |
| | 1942 | 100 | |

FIG. 8.—Deaths of Males. England and Wales.
A. Tuberculosis. Respiratory system.
B. Pneumonia, all forms, ÷ 2.
C. Cancer of lung.

(Data from the Registrar-General's 'Statistical Review of England and Wales for the Year 1942. Tables. Part 1. Medical.')

*Percentage of erroneous diagnoses.*

Some pathologists have emphasized the very high proportion of error in death certificates in general and have produced evidence that the percentage of erroneous diagnoses, as revealed at autopsy, is considerable even in hospitals, and must be greater still in general practice. Thus Willis (1941) found the diagnosis to have been "erroneous or indefinite" in 15 out of 27 cases of cancer of the lung coming to autopsy at the Alfred Hospital, Prahram, Melbourne, and in a larger series (private communication) of 84 autopsies performed by him on cases of pulmonary carcinoma dying in a major teaching hospital, where

TABLE XXIV.—*Analysis of Diagnoses in 84 Cases of Cancer of the Lung Examined at Autopsy (Willis).*

| | | | |
|---|---|---|---|
| Diagnosis correct . . . . . . . | | | 49 = 58 per cent |
| Diagnosis | Tumour, but primary site wrong or uncertain . . | 19 | 35 = 42 ,, |
| | Non-neoplastic disease (mostly tuberculosis, abscess, pneumonia, bronchiectasis) . | 16 | |
| | | | 84 |

there was a special surgical clinic dealing with lung cases, and where full X-ray and bronchoscopic investigation was practised, 42 per cent of the clinical diagnoses were wrong (Table XXIV). Hence the diagnosis of cancer of the lung appears to be still very incomplete, even under favourable conditions, in spite of the great improvements which are generally stated to have been made. At any rate one cannot well emphasize at the same time both the high proportion of cases (e.g. 42 per cent) which escape detection through inaccurate diagnosis, and the adequacy of the improvements in diagnosis to explain the apparent increase in cancer of the lung, though the two statements are of course compatible. Willis does not give any information about false positive diagnoses of pulmonary cancer.

Professor Willis (private communication) has recorded a very instructive example of the statistical effect of diagnostic errors. In a series of 1000 consecutive autopsies which showed the presence of cancer, 155 were found to be cases of cancer of the stomach, and of these, 109, or 70 per cent, had been diagnosed correctly and 46 had therefore received incorrect negative diagnoses. There were also 40 cases diagnosed as cancer of the stomach in which this condition was not present. In this group of 1000 cases, therefore, $109 + 40 = 149$ cases had been diagnosed as cancer of the stomach, and the actual number present was 155. In so far as the total is concerned therefore the clinical answer to the statistical question—how many cases of cancer of the stomach were there in these 1000 cases ?—was very nearly correct. The far greater error in the diagnoses of individuals, of whom 86 were described wrongly, is concealed statistically by the approximate cancellation of the 46 false negatives by the 40 false positives.

This process of attaining accuracy by the balance of positive and negative errors is, of course, neither desirable nor reliable, but it is unavoidable in such material, and is bound to happen to a greater or less extent whether we like it or not. When any decisions are reached on inadequate evidence in any subject, there will be the same tendency for errors in opposite directions to cancel each other.

### FACTORS WHICH MIGHT CAUSE AN INCREASE IN THE DEATHS ATTRIBUTED TO CANCER OF THE LUNG.

The increase in recent years in the number of deaths attributed to cancer of the lung might be due to one, or more than one, of the following factors :

(1) An increase in the actual number of cases of the disease.

It is obviously difficult to obtain any direct evidence of this factor ; one is driven to seek for indirect evidence by means of enquiry how far the second and third factors can be excluded. Some possible causes of a real increase are considered in the next section.

In the earlier paper (Kennaway and Kennaway, 1936) we drew attention to the evidence afforded by cases of cancer of the lung in which an erroneous diagnosis was made based on nervous or mental symptoms due to metastases. Thus Fried and Buckley (1930) record that metastases in the central nervous system were found in 15 of 38 proved cases of cancer of the lung. In 11 of the 15 a diagnosis of primary tumour of the brain was made and the lung tumour overlooked ; an intracranial operation was performed in 11 and the metastases removed in 10 cases. Obviously neither improvement nor fashion in the diagnosis of cancer of the lung (see below) come into play here, and hence if it can be shown that such cases are, as some physicians believe, increasing, a real increase in cancer of the lung is indicated.

(2) Improvement in diagnosis, whereby a larger proportion of the actual number of cases is detected.

The methods of examination of the lungs, and the availability of apparatus for such purposes have undergone very great development in the last twenty years, and it would be remarkable if these changes had not increased the number of cases of cancer of the lung discovered. This matter is discussed under "Errors in diagnosis" above. According to some authorities (e.g. Willis) these improved methods, even when fully available, still leave about half the cases undetected, so we may look for a further considerable increase from this source.

(3) A tendency to identify as cancer of the lung cases which are really of a different nature; that is, a fashion in diagnosis.

We have not found any data which would enable one to judge whether this factor is of any importance; if it is to account for the form of the curve shown in Fig. 1, two or three hundred *more* of such errors must occur every year. Willis (p. 289) does not mention such positive errors in the diagnosis of cancer of the lung.

### SOME POSSIBLE CAUSES OF CANCER OF THE LUNG IN MAN.

Experimental pathology has not yielded any conclusive evidence on this question, as it has done in the case of the occupational cancers of the skin. Most of the laboratory work, such as that of Argyll Campbell, has been done on the mouse, in which species the neoplastic factors in the lung are certainly different from those in man.

(1) In default of experimental evidence, indications of at any rate one direction in which to seek are derived from the material of the General Register Office. These data are compatible with the importance of some factor in town air, e.g. coal smoke.

(*a*) Cancer of the lung differs from that of the upper alimentary tract, larynx and skin in showing no inverse relationship to ascent in the social scale (Stevenson, 1923). Owing to the mixing action of the wind* there is less difference in the outdoor air breathed by different classes than in other social conditions such as food and cleanliness.

(*b*) The higher incidence in towns (Stocks, 1936). Table XXV shows that "there is at every age a steep downward gradient of rates from London through

* An elaborate study of the mixing of air-borne particles was made at Leicester. The effect of wind appears to be not so great as one would expect. "The maximum effect of wind is to displace the point of maximum concentration by ½ mile" (Leicester, 'Atmospheric Pollution,' 1945, p. 123). But it is difficult to believe that, say, an east or west wind blowing across a city does not have a considerable effect in equalizing the condition of the air in the east and west ends. Great importance is attached also to turbulence as a mixing agent. "No exact definition of turbulence has yet been made, but the word has been used, in this report and elsewhere, to describe the readiness with which air mixes with itself. During the last nine months of the Leicester Survey, special attention was given to the measurement of variables connected with turbulence, because of the importance of turbulence in determining the concentration of suspended pollution. It was thought that one of these variables might prove suitable for more general use in connection with measurements of atmospheric pollution" (p. 114). Apparently turbulence includes all forms of disturbance due to unequal local heating.

large and small towns to rural districts." There are, however, exceptional large towns with a low rate (e.g. Bristol). Empirically, smoke concentration in a town is in proportion to the square root of the population (Leicester, 'Atmospheric Pollution,' 1945, p. 124).

(*c*) The low incidence in agricultural workers (Kennaway and Kennaway, 1936). There is an equally low incidence in coal miners.

(*b*) and (*c*) might be explained by the better conditions for diagnosis in urban areas.

Coal smoke does not account well for an *increase* in cancer of the lung during a period when, as one would think, atmospheric pollution has not increased owing to greater use of gas and electricity. The domestic fire is the chief source of soot in the atmosphere,* and it is in the domestic field that the substitution of gas and electricity for coal has been most general.

One must, however, note that ten years ago the authorities on atmospheric pollution were not unanimous on the question whether there had been any measurable diminution.†

TABLE XXV.—*Cancer of Lung. Males. England and Wales.* 1921–1930. (*Stocks.*)

| | Rates per million at ages shown. | | | | | | | | |
|---|---|---|---|---|---|---|---|---|---|
| | 25– | 35– | 45– | 50– | 55– | 60– | 65– | 70– | 75–84. |
| London | 15 | 55 | 121 | 193 | 253 | 263 | 236 | 199 | 215 |
| County Boroughs | 7 | 35 | 73 | 103 | 135 | 168 | 148 | 144 | 93 |
| Other Urban Districts | 5 | 23 | 45 | 77 | 96 | 128 | 150 | 118 | 93 |
| Rural Districts | 7 | 18 | 30 | 34 | 63 | 73 | 87 | 92 | 71 |

(2) One must look, therefore, for some other factor to explain the increase, if there is a real increase, in cancer of the lung in recent years. Many such factors have been suggested, e.g. influenza, tobacco, popular drugs, tarred roads, exhaust gases and other emanations, including lead ethyl, from motor vehicles driven by petrol or Diesel engines.

No attempt is made here to discuss all these possible factors.

* ". . . the percentage of tar in domestic smoke is very high, reaching 30–40 per cent, while in industrial smoke from boiler furnaces and such there is practically no tar " (' Atmospheric Pollution,' 12th Report, p. 46). " A high deposit of tar can only be due to pollution from domestic chimneys or very inefficient industrial furnishings, since in a properly constructed and worked furnace a high temperature is maintained for a sufficient distance from the main place of combustion for all combustible vapours to be fully burned before the furnace gases leave the chimney " (' Atmospheric Pollution,' 23rd Report, p. 9). Soot from the chimney of a dwelling house may contain 40 per cent of tar (Cohen and Ruston, 1925), and the dust suspended in the air of a town (' Leicester Atmospheric Pollution,' 1945) may contain 14 per cent of tar.

† In the ' 22nd Report on the Investigation of Atmospheric Pollution ' (1935–1936, p. 5) the Chairman sums up some rather equivocal evidence thus : " . . . on the whole there has been a definite reduction in the extent of pollution of the atmosphere in Britain during the past twenty years." On p. 11 of the same report, however, the conclusion is drawn that no general statement can be derived from the increases and decreases in different areas during 1925–1935. The special study of atmospheric pollution in Leicester (' Leicester, Atmospheric Pollution,' 1945) showed no difference between the two periods 1927–1932 and 1933–1939 in the amount of tar in deposited matter ; the report makes the suggestion that a decrease in tar might be masked by an increase in material from motor cars. But the inconclusive nature of the data shows that there has been no increase in atmospheric pollution in any way proportional to the increase in cancer of the lung.

(*a*) The subject of tarred roads was dealt with in the earlier paper (Kennaway and Kennaway, 1936).

Although the incidence of lung cancer on those occupations where there is exposure to road dust (see above, Table VI) is rather above the general level, it seems very questionable whether the recent increase of this disease among the general population can be attributed to the tarring of roads. For scores of years before this increase began coal tar was being discharged into the atmosphere in the form of soot by the domestic fire in quantities vastly greater than any that could now be derived from roads. Tarring of the roads has undoubtedly increased the amount of dust derived from tar in the air, but this increase appears negligible in comparison with the amount of soot present already. One sees clouds of smoke drifting over any large town, but one does not see any similar clouds of tar-laden vapour arising continually from tarred roads. The annual consumption of coal for all purposes in Great Britain in 1936–1938 was about 188 million tons, of which 40 to 45 million tons was burned by domestic users, who are by far the greatest producers of smoke and soot.* It is difficult to believe that tarred roads made any important addition to this total.

One must, however, bear in mind that the total amount of soot per unit volume of air may not be the only important factor. The size and physical state of tar particles derived from the domestic fire, and from a tarred road, are no doubt different, and this difference may determine the amount of penetration into the deeper air passages (see, for instance, Boyland, Gaddum and MacDonald, 1947 ; Owens, 1923).

(*b*) *Tobacco*.—A possible connection between tobacco, and especially cigarettes, and cancer of the lung, has been suggested many times, perhaps most recently in the case of Turkey (*Lancet*, 1946, i, p. 435).

Peacock (1943) has pointed out that cancer of the stomach is far more common in man† than, so far as we know, in any other species, and has suggested that this is due to his use of heated foods. A similar argument might be applied in the case of cancer of the lung, which is not known to be prevalent in any of the lower animals. The adenoma of the lung of the mouse, and certain affections of the lung of sheep in South Africa and Iceland, are neoplasms of which the exact nature is uncertain. We know one instance at any rate of the susceptibility of the lung of an animal to a carcinogenic agent, namely the lung of the cat in relation to 2-acetylaminofluorene given by the mouth (Harding, 1946) ; hence there is no reason to think that animals are immune to any such agents. One obvious factor, possibly carcinogenic, to which the lung of man alone is exposed is tobacco smoke.

During the present century very considerable changes have taken place in this country in the social distribution of the various methods of smoking. There were, of course, exceptions to any rule, but roughly one might say that in the earlier part of this period men of the richer classes smoked pipes, cigarettes and cigars, and men of the poorer classes smoked pipes ; cigarette smoking was increasing among the richer women, while women of the poorer classes did not smoke. One never saw a woman scrubbing her front door-step, or hanging out the washing, with a cigarette in her mouth. The great change which has taken

* A. Parker. Chadwick Public Lecture. March 13th, 1945.

† In 1936 in England and Wales, carcinoma of the stomach was assigned as the cause of 7070 out of 27,620 deaths from carcinoma of all sites in males (' Registrar-General's Statistical Review ').

place in the later years is the general increase of cigarette smoking,* and its adoption by women of classes which formerly did not smoke at all. Men in

FIG. 9.—Deaths. England and Wales. 1932–1942.

A. Males } Diseases of coronary arteries, angina pectoris.
B. Females }
C. Males } Cancer of lung.
D. Females }

(Data from the Registrar-General's 'Statistical Review of England and Wales for the Year 1942. Tables. Part 1. Medical.')

FIG. 10.—Consumption in the United Kingdom of tobacco (A) and cigarettes (B).

* The Secretary of State for War stated in the House of Commons on January 23, 1945 (' Parliamentary Debates (Hansard),' **407**, 626) that " In making up the stocks of tobacco and cigarettes for the Army overseas it is assumed that there are nine cigarette smokers to every one pipe smoker. This proportion is based on experience . . ."

occupations which do not subject them to any restrictions in this matter, e.g. builders' workmen, painters, window cleaners, dustmen and road sweepers, who used to smoke a pipe at meal times, now smoke cigarettes while at work. Cigarette smokers are said to inhale more than do smokers of pipes, but it is very difficult to get any conclusive evidence upon this matter, which might be important. There is some American literature, which has been summarized (*Brit. med. J.*, 1946, i, 94) upon the arsenic content of tobacco and tobacco smoke. The occurrence of cancer of the lung in makers of arsenical sheep-dip (p. 285) indicates the possible importance of this factor.

Some writers have contended that smoking cannot be associated with cancer of the lung, because this form of cancer has increased more among men, while the use of tobacco has increased more among women. But the sexual distribution of cancer involves unknown factors (Table XVII), and does not provide a

Fig. 11.—Cigarettes as percentage of tobacco consumed in the United Kingdom, 1924–1943.

very secure basis for an argument of this kind. Of course no claim is made here that the simultaneous increases in the consumption of tobacco, and in cancer of the lung, proves any etiological connection between the two. Other changes which have taken place in the same period, which no one proposes to associate with cancer of the lung, e.g. the increase in the issue of wireless licences, show a very similar curve, and such correlations are a common subject for statistical witticisms. Thus wireless licences have increased at a rate (about 10-fold in the last 20 years) similar to that shown by deaths from cancer of the lung, or from coronary disease (Fig. 9).

The annual consumption of tobacco in the United Kingdom has increased from 128 million pounds in 1924 to 250 million pounds in 1946, and the percentage of these amounts smoked in the form of cigarettes has risen from 56 in 1924 to over 80 in 1943–5* (Fig. 10 and 11). Thus the consumption of cigarettes shows a considerable increase, both absolute and relative.

* We are indebted to Mr. J. R. Willis, M.C., of the Board of Trade for these data. The proportion of cigarettes to total tobacco consumption is available only in those years for which a Census of Production is taken. The monthly net clearances of tobacco mainly by manufacturers for home consumption in the United Kingdom of Great Britain and Northern Ireland are given in the *Monthly Digest of Statistics.*

To summarize the evidence brought together in this section on the incidence of cancer of the lung: the higher mortality in towns, the low mortality in agricultural occupations, and the absence of social gradient, are compatible with a factor in the air, such as coal smoke. In any such comparison of urban and rural areas, the question of facilities for diagnosis must be considered. But coal smoke, which is probably a decreasing contaminant of the air, does not account well for the *increase* of cancer of the lung. Among various possible factors is tobacco smoke ; the consumption of tobacco has risen, and so has the percentage of it smoked in the form of cigarettes, of which the smoke is often inhaled. An effect of tobacco would accord well with the absence of social gradient.

### SUMMARY.

(1) The death certificates for cancer of the lung and of the larynx in males from England and Wales for the years 1921–38 inclusive, numbering 38,418, have been investigated and the periods 1921–32 and 1933–38 are compared. The 63 occupations examined employ about 30 per cent of the male population aged 20 and upwards.

(2) Sources of error in statistical work on death certificates are discussed.

(3) The increase in the recorded cases of lung cancer cannot be attributed to any increase of data obtained by autopsy.

(4) The agricultural and coal-mining industries show a low incidence of cancer of the lung and of the larynx.

(5) A group of open-air occupations, where there is exposure to the dust of roads, has ratios above 100 for cancer of the lung and of the larynx, with the exception that motor drivers have a normal liability to cancer of the larynx. But the comparative incidence of cancer of the lung is not increasing distinctly in any of these occupations, and in the paviours, street masons, concretors and asphalters there has been a distinct fall in the ratio.

(6) The occupations in which there is a liability to silicosis do not show a high incidence of cancer of the lung, but there are in the literature some studies of small numbers of cases in which the two conditions were associated.

(7) Cases of cancer of the lung have occurred in some occupations involving exposure to asbestos.

(8) In the death certificates examined, and in the Reports of the Chief Inspector of Factories, no occupations involving exposure to any kind of dust, except those concerned with asbestos, arsenic and nickel, which employ very small numbers, have been found in which there might be an increased incidence of cancer of the lung.

(9) Workers exposed to coal-gas and tar tend to show an increased prevalence of cancer of the lung, but in the later period studied the incidence does not exceed two-and-a-half times that on the general population.

(10) Occupations concerned with the supply of alcohol have a high incidence of cancer of the larynx.

(11) The later period studied shows a considerable decrease in the occurrence of cancer of the lung in those engaged in the preparation and sale of tobacco.

(12) The very moderate ratio (125) for cancer of the lung in medical men is important in regard to the view that the recent rapid increase in recorded deaths from cancer of the lung is due to the detection of more cases by improved diagnosis, for this is an occupation where the availability of the existing methods for the detection of cancer is presumably at a maximum.

(13) No special occupations have been found, among the 63 examined, to which the increase in the total of cases of cancer of the lung can be attributed. This increase is now so great that the incidence upon any such occupations would have to be very high indeed.

(14) No evidence has been found that tarring of roads has affected the incidence of cancer of the lung. Such data as are available suggest that coal-tar in the atmosphere, whether derived from roads, domestic chimneys or any other source, does not cause an exceptionally high incidence of cancer of the lung. Cotton mule spinners show an especially small liability to cancer of the lung, although they inhale air sprayed with an oil which produces cancer of the skin. Much further work is required on the factors which regulate the penetration of particles and droplets of various shapes and sizes into the air passages.

(15) The higher mortality from cancer of the lung in towns (Stocks), the low mortality in agricultural occupations, and the absence of social gradient (Stevenson) are compatible with an etiological factor in the air such as coal smoke. But in any comparison of urban and rural areas, the question of facilities for diagnosis must be considered.

(16) Soot is probably a decreasing contaminant of the air owing to the substitution of other sources of heat for the domestic fire, which is the chief source of soot-containing smoke. Hence coal smoke does not account well for any recent increase in cancer of the lung. Among various possible factors which have been suggested to account for the increase is tobacco smoke ; the consumption of tobacco has risen, and so has the percentage of it smoked in the form of cigarettes, of which the smoke is often inhaled ; such an effect of tobacco would accord well with the absence of social gradient.

We are indebted to the Registrar-General for the data considered in this paper, and also for kindly permitting us to use some figures which have not yet been made public officially. We have received much assistance in the classification of occupations, and on various other questions, from some members of the staff of the General Register Office, to whom we wish to express our gratitude. We wish to thank Dr. E. R. A. Merewether, Senior Medical Inspector of Factories, for information upon various matters, and especially upon asbestosis, and also Professor R. A. Willis for unpublished material.

We are indebted to the Anna Fuller Fund for secretarial assistance, to the Sir Halley Stewart Trust for a grant, and to Miss Blanche Goode, who has taken part in the classification of death certificates.

### REFERENCES.

NOTE.—No attempt is made in this paper to review the statistical literature relating to cancer of the lung in this or other countries.

ANDERSON, C. S., AND DIBLE, J. H.—(1938) *J. Hyg. Camb.*, **38**, 185.

'Atmospheric Pollution, The Investigation of.' Reports 1–23. London: H.M. Stationery Office.

BOYLAND, E., GADDUM, J. H., AND MACDONALD, F. F.—(1947) *J. Hyg. Camb.*, **45**, 290.

COHEN, J. B., AND RUSTON, A. G.—(1925) 'Smoke. A Study of Town Air.' London.

DIBLE, J. H.—(1934) *Lancet*, ii, 982.

EGBERT, D. S., AND GEIGER, A. J.—(1936) *Amer. Rev. Tuberc.*, **34**, 143.
'Factories, Annual Report of the Chief Inspector of.' London: H.M. Stationery Office.
FRIED, B. M., AND BUCKLEY, R. C.—(1930) *Arch. Path.*, **9**, 483.
GLOYNE, S. R.—(1935) *Tubercle*, **17**, 5.
HARDING, H. E.—(1946) *23rd Ann. Rep. Brit. Emp. Cancer Campaign*, p. 92.
HENRY, S. A.—(1946) 'Cancer of the Scrotum in Relation to Occupation.' Oxford Univ. Press.
*Idem*, KENNAWAY, N. M., AND KENNAWAY, E. L.—(1931) *J. Hyg. Camb.*, **31**, 125.
KENNAWAY, N. M., AND KENNAWAY, E. L.—(1936) *Ibid.*, **36**, 236.
KLOTZ, M. O.—(1939) *Amer. J. Cancer*, **35**, 38.
'Leicester, Atmospheric Pollution in. A Scientific Survey.' London: H.M. Stationery Office, 1945.
LYNCH, K. M., AND SMITH, W. A.—(1935) *Amer. J. Cancer*, **24**, 56.
'Miners' Phthisis Medical Bureau, Report of the, for the 3 years ended July 31, 1935.' Pretoria: Union of South Africa Government Printer, 1936.
MONTGOMERY, H.—(1935) *Arch. Derm. Syph.*, **32**, 218.
*Idem*, AND WAISMAN, M.—(1941) *J. Invest. Derm.*, **4**, 365.
NEUBAUER, O.—(1947) *Brit. J. Cancer*, **1**, 192.
OWENS, J. S.—(1923) *Trans. med. Soc. Lond.*, **45**, 79.
PEACOCK, P. R.—(1943) *Glasg. med. J.*, **139**, 117.
'Registrar-General's Statistical Review of England and Wales.' Tables. Text. London: H.M. Stationery Office.
SEMON, H. C.—(1945) *Proc. Roy. Soc. Med.*, **38**, Section of Dermatology, p. 128.
'Statistical Bulletin,' Metropolitan Life Insurance Co., New York (1939), **20**, 7.
STEVENSON, T. H. C.—(1923) *Biometrika*, **15**, 382.
STOCKS, P.—(1936) *13th Ann. Rep. Brit. Emp. Cancer Campaign*, 239.
WILLIS, R. A.—(1941) *Med. J. Austral.*, Sept. 6, p. 258.

### THE ESTIMATION OF TUMOUR SUSCEPTIBILITY IN PURE LINES.

C. C. SPICER.